# Interaction of the cyclic-di-GMP binding protein FimX and the Type 4 pilus assembly ATPase promotes pilus assembly

**DOI:** 10.1371/journal.ppat.1006594

**Published:** 2017-08-30

**Authors:** Ruchi Jain, Oleksii Sliusarenko, Barbara I. Kazmierczak

**Affiliations:** 1 Department of Medicine (Infectious Diseases), Yale University, New Haven, Connecticut, United States of America; 2 Department of Molecular, Cellular, and Developmental Biology, Yale University, New Haven, Connecticut, United States of America; 3 Department of Microbial Pathogenesis, Yale University, New Haven, Connecticut, United States of America; University of Washington, UNITED STATES

## Abstract

Type IVa pili (T4P) are bacterial surface structures that enable motility, adhesion, biofilm formation and virulence. T4P are assembled by nanomachines that span the bacterial cell envelope. Cycles of T4P assembly and retraction, powered by the ATPases PilB and PilT, allow bacteria to attach to and pull themselves along surfaces, so-called “twitching motility”. These opposing ATPase activities must be coordinated and T4P assembly limited to one pole for bacteria to show directional movement. How this occurs is still incompletely understood. Herein, we show that the c-di-GMP binding protein FimX, which is required for T4P assembly in *Pseudomonas aeruginosa*, localizes to the leading pole of twitching bacteria. Polar FimX localization requires both the presence of T4P assembly machine proteins and the assembly ATPase PilB. PilB itself loses its polar localization pattern when FimX is absent. We use two different approaches to confirm that FimX and PilB interact *in vivo* and *in vitro*, and further show that point mutant alleles of FimX that do not bind c-di-GMP also do not interact with PilB. Lastly, we demonstrate that FimX positively regulates T4P assembly and twitching motility by promoting the activity of the PilB ATPase, and not by stabilizing assembled pili or by preventing PilT-mediated retraction. Mutated alleles of FimX that no longer bind c-di-GMP do not allow rapid T4P assembly in these assays. We propose that by virtue of its high-affinity for c-di-GMP, FimX can promote T4P assembly when intracellular levels of this cyclic nucleotide are low. As *P*. *aeruginosa* PilB is not itself a high-affinity c-di-GMP receptor, unlike many other assembly ATPases, FimX may play a key role in coupling T4P mediated motility and adhesion to levels of this second messenger.

## Author summary

Type IV pili (T4P) are assembled on the surfaces of many bacterial pathogens and commensals through the action of specialized assembly machines whose components and structures are the subject of intense study. Repeated cycles of T4P assembly, attachment and retraction allow bacteria to move or “twitch” along surfaces, efficiently colonize and intoxicate host tissues, and elaborate multicellular structures such as biofilms. Assembly and retraction are powered by specific ATPases, PilB and PilT respectively, but the manner in which their activity is coordinated is still poorly understood. In this work, we provide evidence that a high-affinity c-di-GMP binding protein of *Pseudomonas aeruginosa*, FimX, interacts with the ATPase PilB and promotes PilB-dependent assembly of T4P. Live cell imaging of twitching bacteria shows that FimX localizes to the leading pole of motile *P*. *aeruginosa* and that its recruitment requires both components of the T4P assembly machine and the PilB ATPase. Our work highlights a novel regulatory strategy employed by *P*. *aeruginosa* to control assembly of this broadly conserved virulence factor.

## Introduction

Type IV pili (T4P) are long (5–20 μm), flexible surface structures that perform a variety of functions—motility, adherence, and DNA uptake—in Gram-negative and -positive bacteria, as well as Archaea [1–3]. T4P are capable of generating significant force (30–100 pN) through the rapid retraction of an anchored pilin multimer [4–6]. Recurrent cycles of pilus assembly and disassembly, driven by distinct assembly and retraction ATPases, allow for directed bacterial movement across surfaces. This “twitching motility” is critical for bacterial colonization of surfaces and required for formation and maturation of biofilms by pathogens such as *Pseudomonas aeruginosa* [7].

The machinery required for T4P assembly has been extensively studied in several bacterial organisms, including *P*. *aeruginosa (Pa)*, *Neisseria gonorrhoeae* and *Myxococcus xanthus* [8, 9]. Electron cryo-tomography of the T4a pilus machines of *M*. *xanthus* and *Thermus thermophilus*, and the T4b toxin-coregulated pilus machine of *Vibrio cholerae* has revealed three-dimensional *in situ* structures that are consistent with models proposed for these complexes [10–12]. In each, an outer membrane (OM) secretin (*Pa* PilQ) and pilotin (*Pa* PilF) are required for an emerging pilin multimer to exit the cell envelope. Electron cryo-tomography structures show an inner membrane (IM) protein (*Pa* PilC) aligned with periplasmic rings assigned to *Pa* PilN, PilO and PilP; these three proteins are proposed to form a scaffold that links the outer membrane pore to cytoplasmic components, such as *Pa* PilM [13, 14]. A prepilin peptidase (*Pa* PilD) processes the major pilin (*Pa* PilA) and a number of minor pilins before they can be incorporated into the base of the growing T4P by an assembly ATPase (*Pa* PilB).

Despite this wealth of knowledge about the components of the T4P assembly machinery, it is still not clear how the activity of the assembly (*Pa* PilB) or disassembly (*Pa* PilT and PilU) ATPases is coordinated to achieve net T4P extension or retraction. As the multiple T4P present at a single bacterial pole extend and retract independently of one another [6], regulation must occur at the level of individual ATPase recruitment to or activation at the T4P base. The recent electron cryo-tomography structure of the *M*. *xanthus* type IVa assembly machinery supports this, as PilB and PilT appear to bind in a mutually exclusive fashion to PilC at the T4P base [10].

*P*. *aeruginosa* T4P assembly requires two additional proteins, FimX and PilZ; homologs of these proteins are not present in *M*. *xanthus* or *N*. *gonorrhoeae* [15, 16]. FimX is a degenerate GGDEF/EAL domain protein that binds cyclic-di-GMP via its EAL domain; the purified protein has no diguanylate cyclase activity and exhibits weak phosphodiesterase activity [17–19]. PilZ, despite possessing the characteristic fold of a c-di-GMP receptor, fails to bind c-di-GMP *in vitro* [20]. In *Xanthomonas axonopodis* pv. citri *(Xac)*, PilZ (XAC1133) binds both PilB and FimX (XAC2398), and this complex is proposed to regulate PilB activity [21]. PilZ-FimX binding cannot be demonstrated in *P*. *aeruginosa*, and *Xac* FimX residues implicated in binding *Xac* PilZ are absent in *Pa* FimX [22]. Thus the mechanistic basis for FimX regulation of T4P assembly in *P*. *aeruginosa* remains unknown.

In the present study we investigate the role of FimX in T4P assembly. Using genetic, biochemical and microscopic approaches we report for the first time that FimX interacts with PilB to promote pilus assembly in *P*. *aeruginosa*.

## Results

### FimX localizes to the leading pole of twitching bacteria

**Fig 1:**
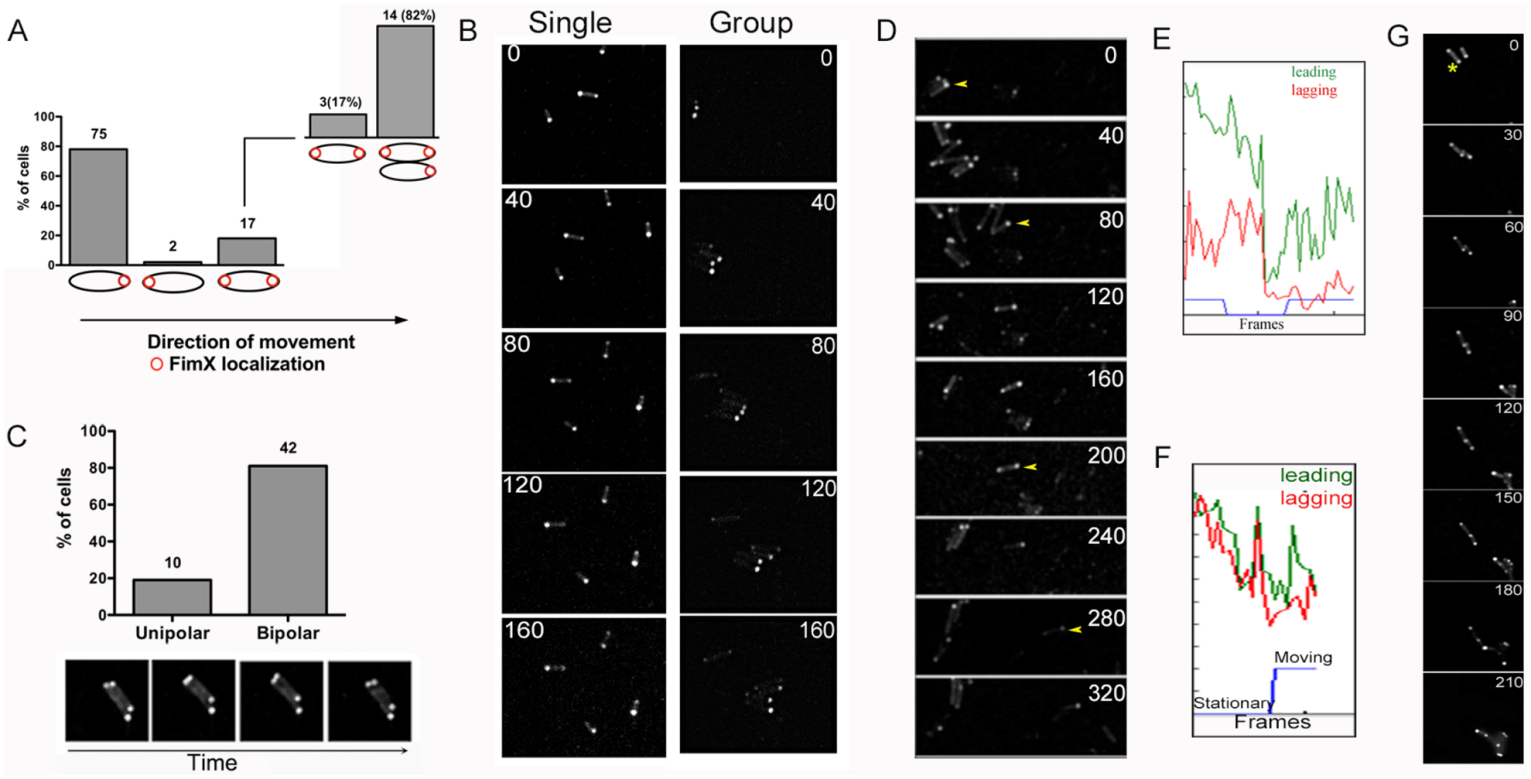
FimX localizes to the leading pole in twitching bacteria. PA103Δ*fimX* ptdimer2-FimX twitching at an agar-glass interface was imaged every 20s by spinning disk confocal epifluorescence microscopy. (A) Location of tdimer2-FimX was scored in 94 moving bacteria. Fourteen of 17 cells with bipolar FimX were in close proximity to moving cells in which FimX localized to the leading pole. (B) Montage of Δ*fimX* ptdimer2-FimX bacteria moving singly or as a group; time lapse interval (s) is indicated for each frame. (C) tdimer2-FimX is predominantly bipolar in stationary cells (n = 52). Lower panel shows a representative time lapse series (every 20s) of cells with bipolar tdimer2-FimX. (D) tdimer2-FimX localization is dynamic and correlated with bacterial movement. A cell (marked with a yellow arrowhead) pauses during imaging (120-240s) before twitching again (280-320s). Fluorescence intensity profile of this cell (E) shows signal at the leading (green) and lagging (red) poles over time of imaging. The blue line indicates a moving (1 or -1) or stationary (0) cell. (F) Bacteria with bipolar FimX appear to be “dragged” by cells with unipolar FimX signal. The fluorescence intensity profile for the cell being pulled (yellow asterisk) is shown in Panel G.

The distribution of FimX in bacteria harvested from agar plates is predominantly unipolar [17]. We monitored subcellular localization of tdimer2-FimX in cells twitching on 1% agar pads, scoring cells as either moving or stationary. In most moving cells (78%), FimX was seen at the leading pole (Fig 1A), confirming the observation by Ni et al. that twitching *P*. *aeruginosa* cells exhibit an asymmetric FimX distribution, with enrichment at the leading pole [23]. This unipolar distribution of FimX was observed in individual moving cells and in groups of twitching bacteria (Fig 1B, S1 Movie). In contrast, 81% of stationary cells showed bipolar localization of FimX (Fig 1C). Upon closer inspection, most twitching cells with bipolar FimX (14 of 17 cells, 82%) or with FimX at the lagging pole, moved only when in close proximity to another cell in which FimX was at the leading pole (Fig 1A, 1F and 1G, S1 Fig, S2 and S3 Movies).

We also observed bacteria alternating between movement and pauses during the course of image acquisition. In these cases, tdimer2-FimX became bipolar when bacteria paused but re-accumulated at the leading pole when movement resumed (Fig 1D and 1E, S4 Movie). Thus, FimX unipolar localization was strongly correlated with directional twitching motility.

### FimX localization is altered by mutation of its cyclic-di-GMP binding domain

**Fig 2:**
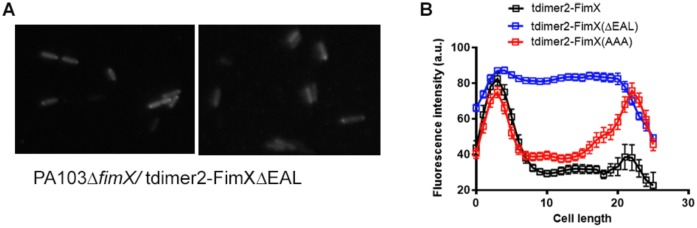
The EAL domain is required for polar localization of FimX. (A) Distribution of tdimer2-FimXΔEAL in PA103Δ*fimX*. Bacteria were imaged on at least 2–3 independent days over 5–6 different fields. The figure shows representative examples. (B) Intensity profiles of tdimer2-FimX, -FimX(AAA), and -FimXΔEAL expressed in PA103 (n = 30/strain). Cell length is shown in pixels.

The carboxy-terminal EAL domain of FimX binds c-di-GMP with sub-micromolar affinity [17, 18, 24]. Deletion of this domain (ΔEAL) or site-directed mutagenesis of the EVL residues in this motif to three alanines (AAA) renders FimX inactive in assays of T4P surface assembly and twitching motility [17]. These mutated alleles of FimX—ΔEAL and AAA—no longer bind c-di-GMP as determined by surface plasmon resonance (SPR) (S2A Fig) [24]. Unlike wild-type FimX, tdimer2-FimX(AAA) appears at both poles when expressed in either wild-type (motile) or Δ*fimX* (non-motile) cells [17]. Deletion of the EAL domain resulted in a third localization pattern, as most tdimer2-FimXΔEAL expressing cells showed diffuse fluorescence (Fig 2). We confirmed that bacteria expressed an intact tdimer2-FimXΔEAL fusion protein by Western blotting (S2B Fig), ruling out degradation as a cause of the diffuse fluorescence signal. Thus, the EAL domain is required for FimX recruitment to or retention at the bacterial pole.

### Polar localization of FimX depends on T4P biogenesis proteins and is affected by mutations in the Pil/Chp chemotaxis system

**Fig 3:**
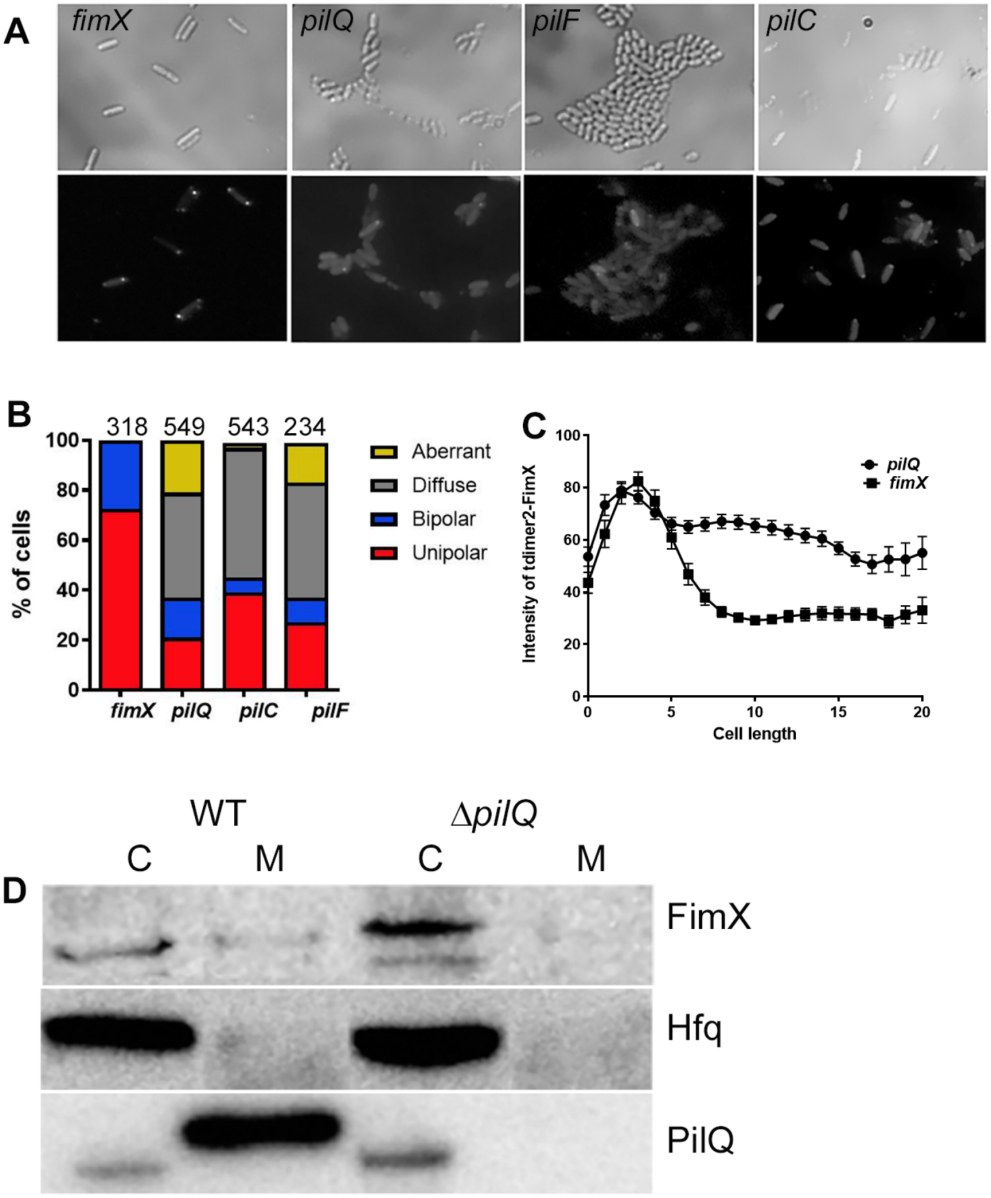
FimX unipolar localization requires structural components of the pilus assembly machinery. (A) tdimer2-FimX localization in live PA14 *fimX*, *pilQ*, *pilF* or *pilC* mutants. Plate grown bacteria were spotted onto 1% agar pads. Upper panel, phase contrast; lower panel, tdimer2 epifluorescence. (B) Distribution of tdimer2-FimX in *fimX*, *pilQ*, *pilF* and *pilC* mutants. The number of cells scored for each strain is noted above each bar. (C) Fluorescence intensity profile of tdimer2-FimX for *fimX* and *pilQ* mutants over the length of the cell in pixels (n = 50). (D) Distribution of endogenous FimX between membrane (M) and cytosolic (C) fractions differs in PA14 vs. *pilQ*::Tn bacteria. Samples were immunoblotted for FimX, the cytosolic protein Hfq, and the outer membrane protein PilQ.

The altered localization of tdimer2-FimX(ΔEAL)—which still has the amino-terminal domain previously shown to be sufficient for polar targeting [15]—prompted us to test whether FimX recruitment to the pole required other protein(s) involved in T4P assembly, using a series of PA14 mutants with mapped Tn insertions. We confirmed that the twitching defect of PA14 *fimX*::Tn was complemented by tdimer2-FimX, and that tdimer2-FimX was predominantly unipolar in this strain (Fig 3A). No significant difference was found in the steady state levels of endogenous FimX in any of these T4P::Tn mutants by Western blotting (S3A Fig).

Disruption of the OM secretin PilQ, the lipoprotein pilotin PilF or the inner membrane protein PilC changed tdimer2-FimX localization from unipolar to predominantly diffuse (Fig 3A, 3B and 3C). Some cells had an unusual distribution pattern of tdimer2-FimX, scored as “aberrant”, in which non-polar signal was detected at the cell periphery. Western blotting confirmed that cells expressed tdimer2-FimX fusion protein of the expected size (S3B Fig).

FimX has no predicted transmembrane domains, but is found in both membrane and cytosolic fractions prepared from wild-type bacteria. This membrane-associated FimX fraction was lost in *pilQ*::Tn bacteria (Fig 3D). We extended these observations by constructing an unmarked, in-frame deletion of *pilQ* in PAO1. Polar tdimer2-FimX localization and FimX membrane association were again lost in the absence of PilQ (S4 Fig). Thus, unipolar localization of FimX requires the presence of the core T4P assembly machinery.

**Fig 4:**
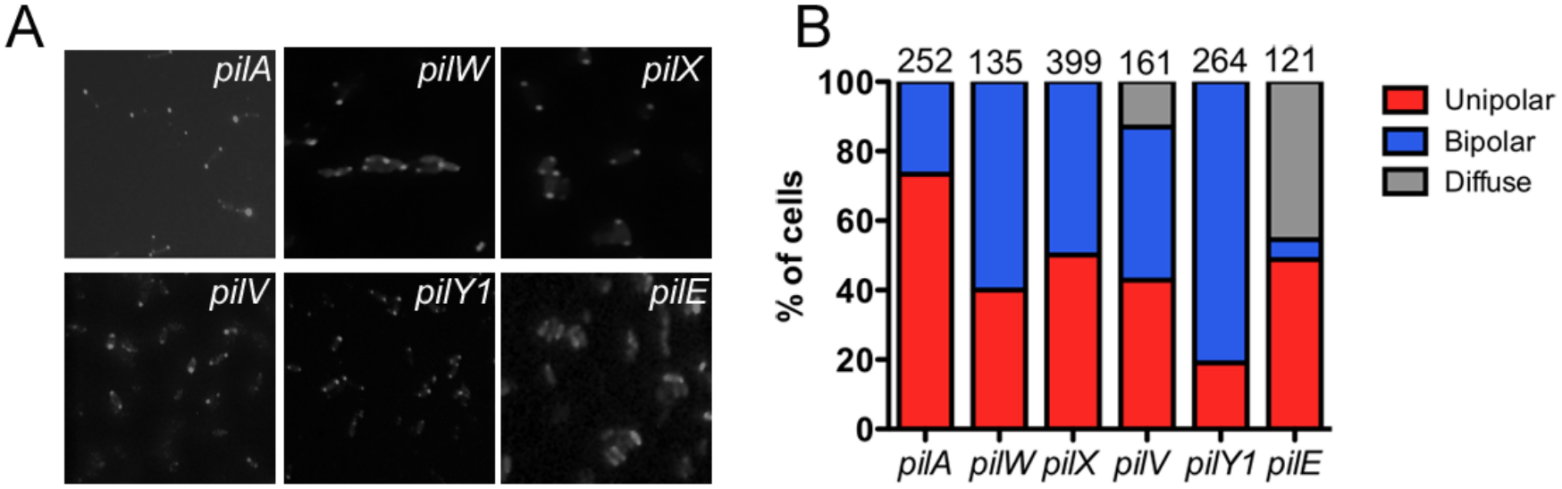
Localization of tdimer2-FimX is altered by the absence of minor pilins and PilY1. (A) The location of tdimer2-FimX in PA14 *pilA* (major pilin), *pilY1*(regulator/adhesin) or *pilV*,*W*,*X*,*E* (minor pilins) mutants was determined by epifluoresence imaging of live bacteria harvested from agar plates. (B) Distribution of tdimer2-FimX expressed in bacteria with transposon insertions in the indicated genes; the number of cells scored for each mutant appears above the bars.

Mutation of the major pilin, *pilA*, did not alter the distribution pattern of tdimer2-FimX as compared to wild-type bacteria (Fig 4). However, disruption of genes encoding a number of the minor pilins (*pilV*, *pilW*, *pilX)* [25] or the regulator *pilY1* [26] did change tdimer2-FimX distribution, with more cells showing a bipolar localization pattern than observed for wild-type bacteria (Fig 4). A third pattern was observed for tdimer2-FimX expressed in *pilE*::Tn bacteria, which lack the minor pilin proposed to couple an assembly priming complex composed of PilVWXY1 to the major pilin subunit, PilA [25]. tdimer2-FimX fluorescence was diffuse in almost 50% of these cells, similar to what was observed in mutants lacking PilQ, PilF or PilC (Fig 4). We could draw no conclusions about FimX localization in the *fimU*::Tn strain, as a tdimer2-FimX fusion protein of the correct size was not detected by Western blotting (S3B Fig). However, we did detect comparable levels of endogenous FimX in PA14 and PA14 *fimU*::Tn, ruling out any obvious effect of FimU on FimX expression or stability (S3A Fig).

**Fig 5:**
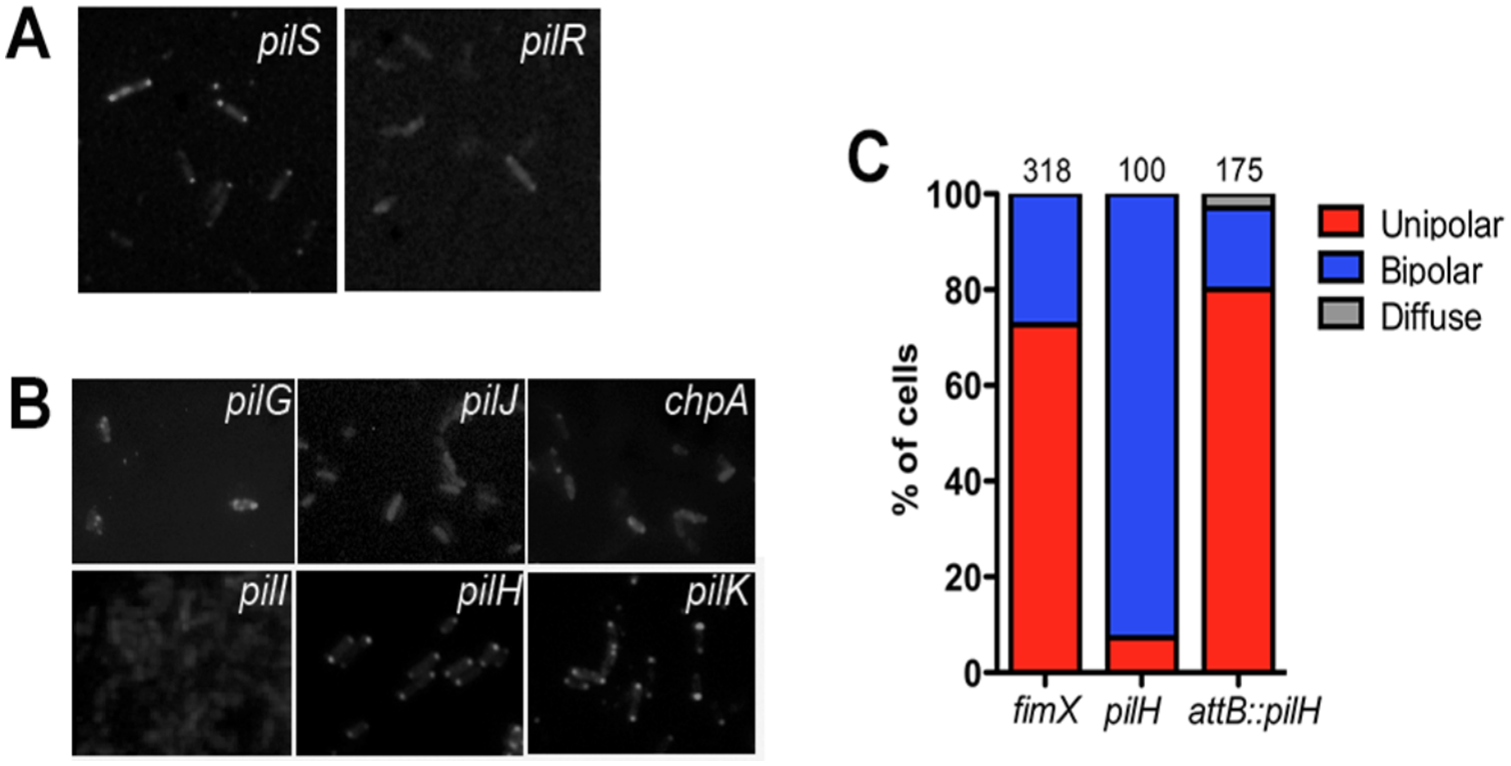
FimX distribution is altered in mutants lacking T4P regulatory proteins. tdimer2-FimX distribution in bacteria lacking (A) the PilS/PilR TCS and (B) Pil/Chp chemotaxis cluster proteins was determined by epifluoresence imaging of live bacteria. (C) Localization of tdimer2-FimX in PA14 *pilH*::Tn and WT *pilH* complemented strain. The *pilH* gene was introduced at the *attB* site on the chromosome and expressed from its endogenous promoter.

We also investigated FimX localization in mutants lacking regulatory proteins required for T4P protein expression and assembly. *P*. *aeruginosa* has a two-component system that regulates expression of proteins involved in T4P biogenesis [27]. Disruption of the sensor kinase PilS did not alter tdimer2-FimX localization, while mutation of the PilR response regulator resulted in a dim and diffuse tdimer2-FimX signal (Fig 5A). Western blotting showed a band migrating at the predicted molecular weight of tdimer2-FimX in *pilR*::Tn bacteria (S3B Fig), and steady-state levels of endogenous FimX appeared to be unaffected by this mutation (S3A Fig).

A marked shift in FimX localization was observed in mutants in the Pil/Chp chemotaxis system, which links cAMP signaling with T4P motility [28]. Both *pilH*::Tn and *pilK*::Tn showed bipolar localization of tdimer2-FimX in >90% and 70% of cells, respectively (Fig 5B). Unipolar tdimer2-FimX localization was restored by complementing the *pilH*::Tn mutant with *pilH* under its own promoter integrated at the chromosomal *attB* site (Fig 5C). Bipolar tdimer2-FimX localization was also observed in PAO1Δ*pilH*, which carries an unmarked, in-frame deletion of *pilH* (S5 Fig). Mutation of PilK (CheR methyltransferase homolog) has no effect on T4P motility or surface pilin [29], while PilH mutants (CheY-like response regulator) exhibit altered T4P motility [30, 31]. In contrast, FimX did not localize to the pole in Pil/Chp transposon insertion mutants reported to lack both surface-associated pilin and twitching motility [28]. Specifically, the tdimer2-FimX signal was diffuse and dim in strains with Tn insertions in *pilJ* (methyl accepting chemotaxis protein receptor), *chpA* (putative histidine kinase) and *pilI* (*CheW* adaptor protein homologue) (Fig 5B). tdimer2-FimX fluorescence in *pilG* (*CheY* like response regulator) [32] differed somewhat, with some bacteria showing a faint, “aberrant” localization pattern while most exhibited no fluorescence (Fig 5B).

### FimX localization is influenced by the presence of assembly and retraction ATPases

**Fig 6:**
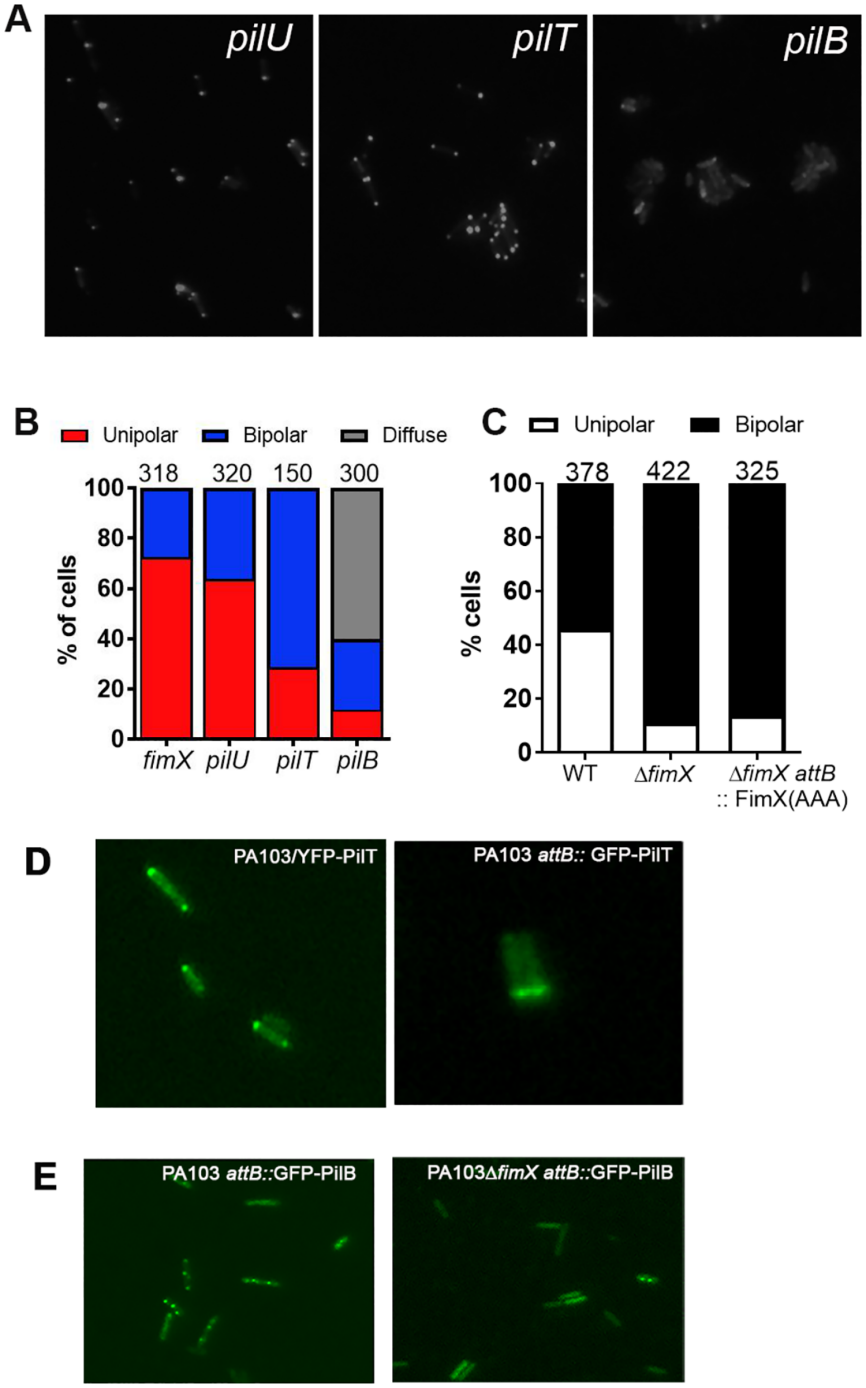
FimX and PilB localization depend on each other’s presence. (A) tdimer2-FimX localization in ATPase mutants was determined by epifluoresence imaging of live bacteria. (B) Distribution of tdimer2-FimX localization is shown for each mutant; numbers above each bar indicate how many cells were scored. (C) Localization of YFP-PilT in PA103, PA103Δ*fimX* and PA103Δ*fimX attB*::*FimX(AAA)*. The number of cells scored for each strain is shown above the corresponding bar. (D) Fluorescence images of PA103 bacteria expressing plasmid borne YFP-PilT (left) or single copy *attB*:: P_*pilT*_-GFP-PilT. (E) Fluorescence images of PA103 and PA103Δ*fimX* bacteria expressing single-copy *attB*::P_*pilB*_-GFP-PilB.

*P*. *aeruginosa* encodes two retraction ATPases, PilT and PilU, which have different phenotypes when mutated [33]. Δ*pilT* bacteria do not twitch and are resistant to the pilus-specific phage PO4; Δ*pilU* cells exhibit greatly reduced twitching motility but are still PO4 sensitive [34]. tdimer2-FimX localization in *pilU*::Tn bacteria was indistinguishable from that observed in wild-type cells. In contrast, most (>80%) *pilT*::Tn bacteria had bipolar tdimer2-FimX fluorescence (Fig 6A and 6B), similar to what we observed for *pilH*::Tn and *pilK*::Tn bacteria. A third localization pattern was observed for *pilB*::Tn bacteria: these cells had a diffuse, cytosolic distribution of tdimer2-FimX, like that observed for mutants lacking the PilC, PilQ or PilF T4P machinery components (Fig 6A and 6B).

Given the strong FimX localization phenotypes associated with *pilB* and *pilT* mutants, we also tested whether the distribution of these ATPases was altered in the absence of FimX. We expressed plasmid-encoded YFP-PilT in both PA103 and PA103Δ*fimX* bacteria, confirming that this construct complements Δ*pilT* bacteria for twitching and has no dominant negative effect on motility of wild-type bacteria [33]. In about 50% of wild-type bacteria, YFP-PilT localized to one pole, while in the remainder of cells YFP-PilT was bipolar (Fig 6C and 6D). A P_*pilT*_-GFP-PilT construct was also integrated into chromosomal *attB* site of PA103, and showed a similar localization pattern (Fig 6D). In Δ*fimX* bacteria, however, we observed a change in YFP-PilT localization, with >90% of bacteria showing bipolar fluorescence (Fig 6C). Furthermore, the FimX(AAA) allele that cannot bind c-di-GMP phenocopied Δ*fimX*, despite the fact that FimX(AAA) itself has a bipolar distribution (Fig 2B). Thus FimX directly or indirectly affected PilT localization, but only when its c-di-GMP binding site was intact.

Plasmid-borne YFP-PilB was previously used in studies of PilB localization, which reported a unipolar distribution for this protein [33], but this construct does not complement Δ*pilB* motility and has a dominant negative effect on twitching of wild-type cells (S6A Fig). We therefore integrated a P_*pilB*_-GFP-PilB construct at the chromosomal *attB* site and confirmed that bacteria carrying this extra *GFP-pilB* gene had no twitching defect (S6B Fig). This single copy construct showed a very different subcellular distribution pattern in wild-type bacteria, with GFP-PilB present at both polar and non-polar sites (Fig 6E). GFP-PilB distribution changed in Δ*fimX* bacteria: fluorescence signal was lost from the poles and appeared throughout the cytoplasm (Fig 6E). Thus FimX affects the subcellular distribution of both PilT and PilB, and is required for polar GFP-PilB localization.

### FimX acts by promoting PilB-dependent assembly and not by inhibiting PilT-mediated retraction

**Fig 7:**
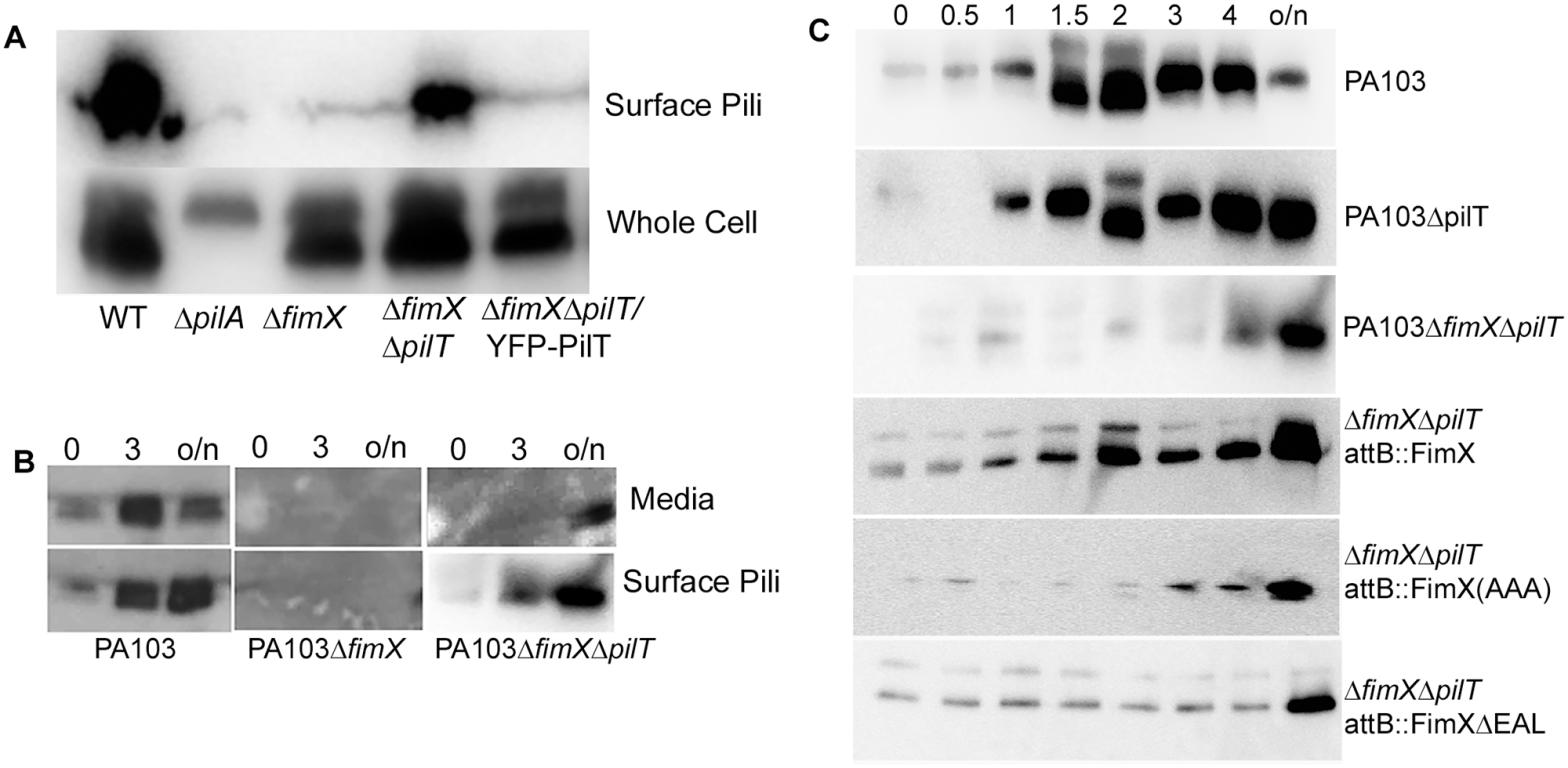
FimX promotes pilus assembly rather than inhibiting pilus retraction. (A) Western blot with anti-PilA antiserum of whole cell extract and sheared surface-associated pilin from PA103 and isogenic deletion mutants after overnight culture. Samples were normalized to the amount of protein in the total cell extract. (B) Bacterial cells were vortexed (to shear off surface pili), pelleted and resuspended in fresh media. Cell-associated pili (Surface Pili) and spent media (Media) were collected for indicated strains immediately after resuspension (t = 0h), 3h after resuspension, and after overnight (o/n) culture. Surface pili and TCA-precipitated spent medium were separated by 15% SDS-PAGE and immunoblotted with anti-PilA antiserum. (C) Bacterial cells were vortexed to shear off surface pili, resuspended in fresh medium, and collected at specified intervals after resuspension (0.5h, 1h, 1.5h, 2h, 3h, 4h and overnight (o/n)). Surface pili were then sheared from bacteria, separated by 15% SDS-PAGE and immunoblotted for PilA protein.

Given the interdependence of FimX, PilB and PilT localization patterns in *P*. *aeruginosa*, we hypothesized that FimX increases net surface T4P assembly by promoting PilB-dependent assembly and/or by inhibiting PilT-dependent retraction. In order to distinguish between these possibilities, we constructed a PA103Δ*fimX*Δ*pilT* mutant and examined surface pilin levels in this strain. In contrast to Δ*fimX* bacteria, the Δ*fimX*Δ*pilT* mutant did assemble pili, and this phenotype was reversed by expressing YFP-PilT (Fig 7A, S7 Fig). We controlled for the possibility that pili were also assembled by Δ*fimX* bacteria, but somehow more susceptible to shearing or shedding from cells, by measuring pilin present in media as well as surface-associated pilin on harvested cells. In wild-type cultures, we observed accumulation of pilin in both the media and bacterial surface-associated fractions over time (Fig 7B). In Δ*fimX* bacteria, pilin was detected in neither media nor surface-attached fractions. We confirmed that assembly of surface pili still required PilB, as we detected no surface pilin assembly by PA103Δ*fimX*Δ*pilT*Δ*pilB* bacteria (S8 Fig).

If Δ*fimX*Δ*pilT* bacteria can assemble T4P, PilB must retain activity in the absence of FimX. Δ*fimX* bacteria could fail to assemble pili either because PilB activity is diminished, or PilT activity is increased, in the absence of FimX. We distinguished between these possibilities by comparing the kinetics of surface pilus assembly in wild-type, Δ*pilT* and Δ*fimX*Δ*pilT* bacteria. Surface pili of exponential-phase cells were sheared at t = 0, and surface assembly of new T4P was monitored over time. Surface pilin was detected within 60 minutes for WT and Δ*pilT* bacteria, but took 3–4 hours to appear for Δ*fimX*Δ*pilT* bacteria (Fig 7C). As PilT’s absence is not sufficient to restore normal kinetics of T4P assembly in the double mutant, FimX must promote the activity of PilB. Complementation of Δ*fimXΔpilT* bacteria with FimX restored wild-type kinetics of surface pilin assembly, but neither the FimX (AAA) nor FimXΔEAL proteins were able to rescue surface pilin assembly in this assay (Fig 7C). Thus, we conclude that FimX does not function to stabilize assembled pili, nor to inhibit their retraction, but instead favors PilB-dependent assembly of T4P, either by increasing the number of assembling pili and/or the net duration or rate of assembly.

### FimX mutants that cannot bind cyclic-di-GMP do not interact with PilB

**Fig 8:**
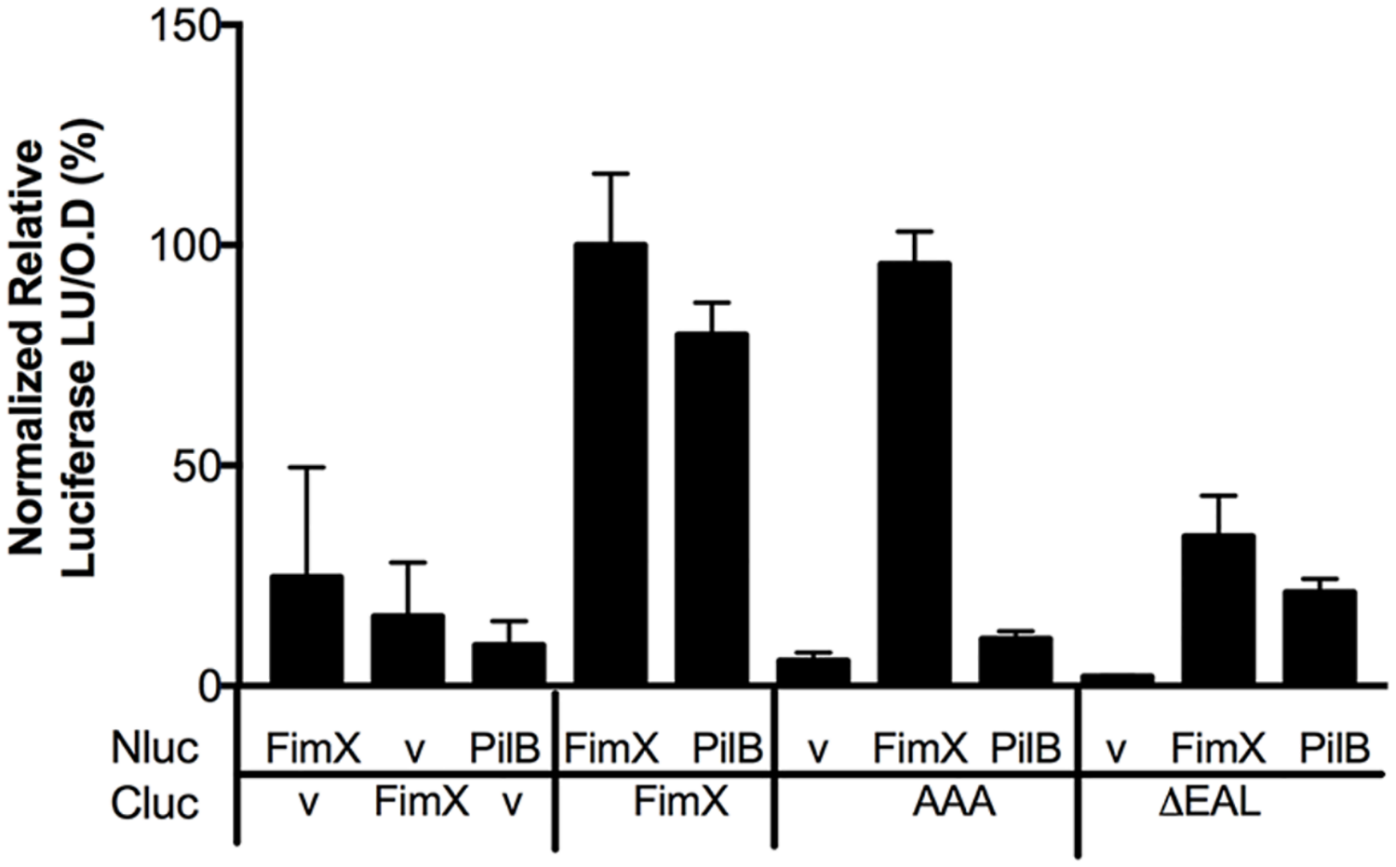
PilB and FimX interactions are seen in a split luciferase complementation assay. PA103 co-expressing Nluc- and Cluc-fusion protein constructs as indicated (or empty vector “V”) were grown overnight on LB plates containing appropriate antibiotics and 0.05% arabinose. Bacteria were harvested by scraping, resuspended in PBS and normalized by OD_600_. RLU (luciferase units/OD_600_) was measured on addition of 1 μM coelenterazine substrate to the bacterial cells. Bars represent the mean ± SD of two technical replicates from a representative experiment. Values for FimX-FimX are set to 100%.

As FimX appeared to promote PilB activity directly or indirectly, we tested whether these two proteins interacted. We adapted the split-luciferase complementation assay [35] to monitor protein-protein interactions in *P*. *aeruginosa* and *E*. *coli* cells. Plate-grown *P*. *aeruginosa* expressing NLuc- and CLuc-fusions to FimX, PilB or PilT were assayed for luciferase activity; FimX, which forms a homodimer [18], served as a positive control in these assays. We observed luminescence for bacteria expressing NLuc-PilB and CLuc-FimX. PilB did not interact in this assay with either FimXΔEAL or FimX(AAA) (Fig 8). We also did not observe any interaction between FimX and PilT in this assay (S9 Fig).

In *Xanthomonas*, a three-way interaction between FimX, PilB and the T4P biogenesis protein PilZ has been reported [21]. We expressed NLuc-PilB and CLuc-FimX in Δ*pilZ* bacteria and continued to observe a strong interaction between FimX and PilB (S9 Fig). This observation was consistent with prior reports that *P*. *aeruginosa* PilZ and FimX do not interact *in vitro* [22]. To test whether the PilB-FimX interaction required other *P*. *aeruginosa* proteins, we introduced these split luciferase constructs into *E*. *coli*, which does not encode Type 4 pili. We observed luciferase activity when NLuc-PilB was expressed with CLuc-FimX, but not with CLuc-FimX(AAA) (S10 Fig). This suggests that the interaction between FimX and PilB is direct, although it may be promoted or stabilized by the presence of other T4P assembly proteins.

We also tested whether purified FimX and PilB interact. PilB forms a hexameric assembly [36], which complicated the interpretation of isothermal calorimetry and surface plasmon resonance experiments. We therefore used size exclusion chromatography (SEC) to examine the migration of FimX and PilB individually and after co-incubation. As FimX purified bound to c-di-GMP, this nucleotide was added to all samples to control for its presence.

**Fig 9:**
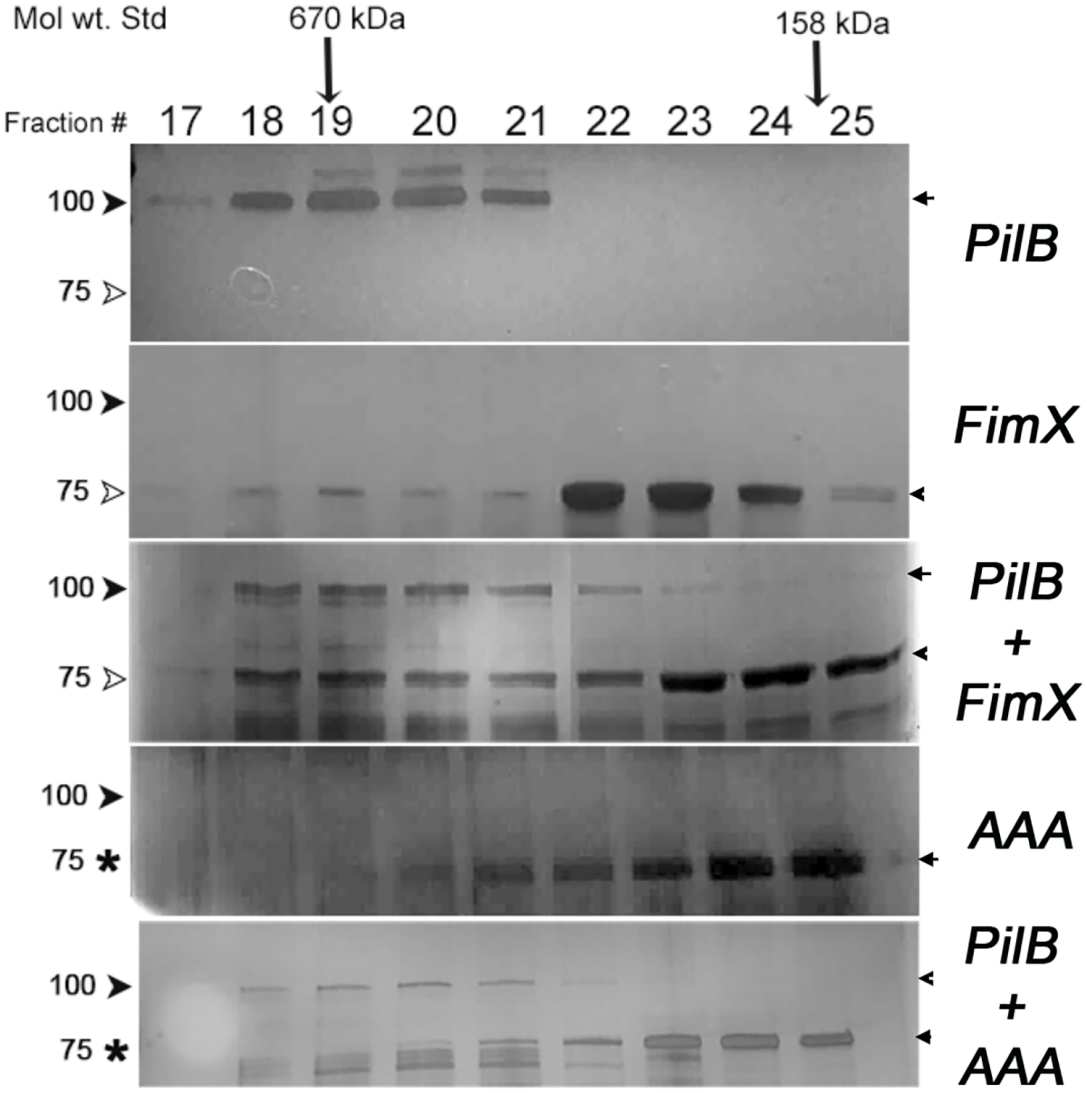
The elution profile of FimX, but not FimX(AAA), is altered by co-incubation with PilB. PilB, FimX and FimX(AAA) were analyzed by size exclusion chromatography individually and after co-incubation, as indicated. Collected fractions (Fraction 17–25) were separated by SDS-PAGE and visualized by staining. The positions at which the molecular weight standards thyroglobulin (670 kDa) and γ-globulin (158 kDa) eluted are marked by arrows.

When analyzed individually, 90% of FimX eluted as a peak with an apparent molecular weight of ca. 150 KDa, consistent with its proposed structure as a dimer in solution [18], while 100% of PilB eluted at an apparent molecular weight of ~670 KDa. The elution profile of FimX changed when it was pre-incubated with PilB: 34% of FimX now co-eluted with PilB (fractions 18–21) (Fig 9). Purified FimX (AAA) showed a similar elution profile to FimX when analyzed alone; however, co-incubation with PilB had not effect on the elution profile of FimX(AAA) (Fig 9). Thus two lines of experimental data indicate that PilB and FimX interact directly, and that FimX must be able to bind c-di-GMP for this interaction to occur.

## Discussion

T4P allow bacteria to adhere to surfaces and to other bacteria, and power surface-associated motility through cycles of extension and retraction. Single cell visualization and tracking reveal that T4P allow *P*. *aeruginosa* to crawl, “cartwheel” and even catapult off surfaces, behaviors that contribute to surface exploration and colonization [37–40]. Theoretical models suggest that directionally persistent crawling of these rod-shaped bacteria is more likely when T4P assembly and retraction occur at one pole [41], as has been experimentally observed for both *P*. *aeruginosa* and *M*. *xanthus* [6, 42]. Yet, the machines that assemble T4P are seen at both poles of *M*. *xanthus* [10]—and components of these machines localize to both poles of *P*. *aeruginosa* [43]—raising the question of how assembly is restricted to one pole at a time. In *M*. *xanthus*, the Frz chemosensory system controls which pole is leading or lagging, and can reverse the direction of motility [44]. Much work has focused on proteins that act downstream of Frz to either recruit PilB and PilT to the same bacterial pole (e.g. the GTPase SofG and its cytoskeletal interaction partner BacP), or to sort PilB and PilT to the leading versus lagging poles, respectively (e.g. the response regulator RomR, the small GTPase MglA, and the latter’s activator, MglB) [45, 46]. Despite the progress made in understanding how these proteins dynamically establish cell polarity, it is still not clear whether physical separation of PilB and PilT is the primary mechanism by which extension and subsequent retraction of T4P are controlled in *M*. *xanthus*. Much less is known for *P*. *aeruginosa*, which has recently been shown to modulate reversals of twitching motility for effective chemotaxis toward attractants [31].

By tracking *P*. *aeruginosa* cells twitching at a glass-agar interface we have demonstrated that FimX, a c-di-GMP binding protein required for twitching motility [15, 17], localizes to the leading pole of moving bacteria, where T4P assembly and retraction are occurring. The strong correlation between a unipolar FimX signal and motility of individual bacteria suggested that FimX might be involved in the regulation of T4P assembly and/or retraction, and prompted us to investigate the requirements for FimX localization further. A visual screen carried out in mutants of T4P assembly machine proteins revealed that FimX was not localized to the pole when the OM secretin PilQ, the lipoprotein PilF, the inner membrane protein PilC, or the PilB assembly ATPase were absent. It is not expected that FimX, a cytoplasmic protein, interacts with PilQ or PilF directly. In *M*. *xanthus*, however, the PilQ OM secretin is proposed to initiate assembly of the Type 4 pilus machine (T4PM) in an outside-in manner, recruiting a periplasmic/IM complex composed of PilP, PilO and PilN that in turn recruits the IM PilC protein and the cytoplasmic PilM protein [47]. Many of the individual protein-protein interactions implicated in this assembly process have been demonstrated for *Pa* homologs [48, 49], and PilO localization to the *Pa* poles *in vivo* requires PilQ and PilP [43]. Thus FimX delocalization in the absence of these T4PM proteins suggests that FimX is recruited to and/or retained at the assembly machine.

Unipolar FimX localization further required the assembly ATPase PilB. The *M*. *xanthus* T4PM can be assembled in the absence of PilB [47], and “empty” machines lacking assembling T4P are observed in both Δ*pilB* and Δ*pilB*Δ*pilT* cells [10]. PilB and PilT appear to interact with the assembled machine via a cytoplasmic “dome” at its base, proposed to consist of PilC’s N-terminal and C-terminal cytoplasmic domains [10]. Purified *Pa* PilB and the *Pa* PilC N-terminal domain have been shown to interact *in vitro*, while genetic data suggests that *Pa* PilC’s C-terminal domain is involved in T4P retraction, perhaps through interactions with PilT and/or PilU [50]. Accordingly, the atomic structures of PilB and PilT can be modeled into the cytoplasmic dome of the *M*. *xanthus* T4PM in such a manner that they contact the N- and C-terminal domains of PilC, respectively; only one hexameric ATPase can be accommodated at a time [10].

Our finding that FimX localization depends on PilB could indicate that FimX interacts with the T4PM only when PilB is bound to it, or that FimX and PilB are recruited to the T4PM together. Our observation that GFP-PilB localization is altered in Δ*fimX* cells is consistent with either model, particularly if FimX can influence PilB retention at the T4PM. *Pa* PilB localization may differ from that reported for *M*. *xanthus*, where bipolar PilB is observed by immunofluorescence in fixed bacteria lacking other T4PM proteins, suggesting that PilB trafficking to the pole is independent of T4PM proteins in *M*. *xanthus* [47]. This may reflect the presence of a network of small GTPases and cytoskeleton proteins that control PilB and PilT localization in *M*. *xanthus*, establish cell polarity, and coordinate T4P dependent motility with gliding motility [45].

The apparent dependence of FimX and PilB localization on each other’s presence in *P*. *aeruginosa* prompted us to ask whether these proteins interact *in vivo*. We tested this by assaying FimX interactions with PilB via split-luciferase complementation, and observed a strong luciferase signal when fusion proteins were expressed in either wild-type or Δ*pilZ* bacteria. We also observed luminescence when FimX and PilB fusions were expressed in *E*. *coli*, indicating that no other *P*. *aeruginosa* proteins were required for this interaction. Similar experiments carried out with FimX and PilT did not demonstrate an interaction between these proteins in *P*. *aeruginosa* or *E*. *coli*. Size-exclusion chromatography elution profiles of purified FimX and PilB, assayed individually or after co-incubation, provided further evidence of a direct FimX-PilB interaction. Thus regulation of T4P assembly in *P*. *aeruginosa* appears to differ from that proposed for *X*. *axonopodis*, where PilZ binds to PilB and to FimX and is proposed to mediate their interactions.

The phenotypes of Δ*fimX* bacteria—absent surface pilin and motility—could result from decreased T4P assembly, increased T4P retraction, or increased loss of assembled T4P from bacteria. Our genetic analysis of the kinetics of T4P assembly discriminates between these possibilities and demonstrates that FimX acts by increasing the net rate of PilB-dependent T4P assembly. We speculate that this could occur via several mechanisms. FimX could increase the probability of PilB binding at a T4PM, either by facilitating its recruitment to the T4PM or by prolonging its retention at the T4PM, results that are consistent with our *in vivo* localization data. FimX could also increase the ATPase activity of PilB directly; however, we have not been able to demonstrate this with purified PilB and FimX proteins *in vitro* (S11 Fig). Lastly, a FimX-PilB interaction could modulate the accessibility of PilB to other regulators of T4P dynamics, such as the Pil/Chp chemotaxis proteins.

FimX binds c-di-GMP with high affinity (K_D_ ~ 90 nM) via its EAL phosphodiesterase domain and becomes largely c-di-GMP bound when *P*. *aeruginosa* transitions from liquid to surface-associated growth, as intracellular [c-di-GMP] increases from <50 nM to ~300 nM [51–53]. FimX dimerization, which is modeled to occur via interactions of the amino-terminal REC domain, is insensitive to the presence/absence of c-di-GMP, but long-range conformational changes have been reported in the REC and adjacent linker domains upon c-di-GMP binding [18, 24]. FimX alleles that no longer bind c-di-GMP, such as FimX(AAA), do not support T4P assembly or twitching motility [17] and no longer interact with PilB by any of our assays. Thus FimX, by virtue of its ability to bind c-di-GMP with high affinity, may serve to “license” pilus assembly at the lower intracellular c-di-GMP concentrations that bacteria experience after surface attachment, leading to directionally persistent movement and surface colonization [52]. Under conditions of high c-di-GMP, such as those seen in rugose small colony variants or biofilms, FimX is dispensable for T4P assembly—but T4P are assembled at non-polar sites, twitching zones are decreased, and adhesion—rather than motility—may be key [52].

Even though T4P and their biogenesis proteins have been extensively studied for decades in *P*. *aeruginosa* [54], surprises still remain. Tracking of *P*. *aeruginosa* twitching at the single cell level in a novel microfluidics system has recently revealed that *P*. *aeruginosa* reverses direction during twitching motility in order to bias net movement toward a chemoattractant source [31]. Surprisingly, Pil/Chp mutants previously defined as nonmotile (e.g. *pilG*) or as having reduced motility (e.g. *pilH*) in plate-based stab assays of motility [32, 34] showed normal (*pilG*) or much increased (*pilH*) twitching speed and aberrant reversal behavior/persistence when tracked as single cells within a chemoattractant gradient [31]. The altered localization patterns observed for FimX in Pil/Chp mutants (Fig 5) in our study may indicate that FimX, along with PilB and PilT, is subject to regulation by this chemotaxis system.

In many organisms that lack FimX homologs, c-di-GMP has emerged as an important regulator of pilus assembly. Recent papers demonstrate that the assembly ATPase for the *Vibrio cholerae* adhesive MshA type 4 pilus is a receptor for c-di-GMP (Kd ~ 2 μM) [55, 56], as is the PilB2 T4P assembly ATPase of *Clostridium perfringens* [57]. These specific examples represent a newly described c-di-GMP binding domain, “MshEN”, present in diverse bacterial proteins including many ATPases associated with T4P assembly and Type 2 secretion systems [58]. Although the ATPases associated with T4P assembly/retraction in *V*. *cholerae* (TcpT (VC0835), PilB (VC2424)) and *P*. *aeruginosa* (PilB, PilT, PilU) do not bind c-di-GMP [56], the PilB protein of *M*. *xanthus* is predicted to do so [58]. We can only speculate that FimX, a high-affinity c-di-GMP binding protein, allows *P*. *aeruginosa* to assemble polar T4P when intracellular c-di-GMP is relatively low, in the absence of a PilB protein with high c-di-GMP affinity.

## Materials and methods

### Strains and growth conditions

Bacterial strains and plasmids used in this study are listed in S1 Table. Both *E*. *coli* and *P*. *aeruginosa* were cultured in Luria Broth (LB) medium with antibiotics as required at the following concentrations: gentamicin, 15 μg ml^-1^ for *E*. *coli* or 100 μg ml^-1^ for *P*. *aeruginosa*; ampicillin, 100 μg ml^-1^ for *E*. *coli*; carbenicillin, 200 μg ml^-1^ for *P*. *aeruginosa*; tetracycline, 20 μg ml^-1^ for *E*. *coli* or 100 μg ml^-1^ for *P*. *aeruginosa*. Commercial chemically competent *E*. *coli* was used for transformations (Invitrogen) and *P*. *aeruginosa* was transformed by electroporation [59]. All bacterial strains were stored at -80°C as 15% (v/v) glycerol stocks.

### Strain construction

All PCR primers employed in this study are listed in S2 Table and were synthesized by the Keck Facility (Yale University). A collection of PA14::Tn mutants in T4P biogenesis genes used in this study were obtained from Dr. George O’Toole (Dartmouth University). (S1 Table). These strains were electroporated with pUCP carrying tdimer2-FimX expressed from the *fimX* promoter, and transformants selected on LB carbenicillin.

Unmarked, in-frame deletions of selected genes (*pilB*, *pilT*, *pilH*, *pilQ*, *pilZ*) were constructed in PA103, PAO1, PA103Δ*fimX* and/or PA103Δ*fimX*Δ*pilT* (as specified) via allelic exchange. DNA immediately upstream and downstream of the specific gene was amplified from WT genomic DNA with Phusion polymerase (NEB), using gene specific N1, N2 (upstream) and C1, C2 (downstream) sets of primers. Amplification products were cloned in tandem into the *sacB*-containing pEX18-Gm^R^ plasmid. This plasmid was transformed into *E*. *coli* S17-1 and mobilized into *P*. *aeruginosa* by mating. Merodiploid ex-conjugants were selected on VBM gentamicin, then streaked to VBM plus 10% sucrose to select for a second recombination event. Gm^S^/Suc^R^ candidates were isolated and screened by PCR for successful deletion of the selected gene.

PilB and PilT were expressed as GFP tagged proteins under their own promoters at the *attB* site on the chromosome. The final clone was constructed by Gibson Assembly (New England Biolabs) and includes 500 bp upstream of the indicated gene (amplified using PilB or PilT G1 and G2 primers), followed by GFP (amplified using PilB or PilT G3 and G4 primers), a flexible linker (GGAAGG) and the full-length gene of interest (PilB or PilT using G5 and G6 primers), cloned into KpnI/EcoRI (PilB) or KpnI/BamHI (PilT) sites in miniCTX-2. The resultant constructs, GFP-PilB or GFP-PilT, were integrated at the *attB* site of *P*. *aeruginosa* and vector backbone sequences removed by Flp recombinase [60].

A 1kb region including the promoter, the upstream region and the coding sequence of *pilH* was amplified using the primer pair PilHupF and PilHR. The resultant amplicon was cloned in EcoR1/HindIII sites in miniCTX2 and confirmed by sequencing. The fragment was integrated at the chromosomal *attB* site in *P*. *aeruginosa* PA14 *pilH*::Tn and PAO1Δ*pilH* by mating followed by excision of the vector backbone by Flp recombinase [60].

PilB (or PilT) was amplified from PA103 using F and R primers. The amplicon was recombined in a Gateway expression vector, pVL847 (provided by Dr. Vincent Lee) that resulted in 10XHis-MBP tagged recombinant PilB (or PilT). These constructs were transformed into *E*. *coli* BL21(DE3) for expression and purification.

### Protein expression and purification

For purification of 10x-His-MBP tagged PilB (or PilT), the cultures were grown at 37°C in LB (with appropriate antibiotic) to OD_600_ ~ 0.4–0.6 and induced with 0.4 mM IPTG for 3 hrs. Cells were harvested and resuspended in a 1/50 culture volume of lysis buffer (50 mM Phosphate buffer pH 7.5, 500 mM NaCl, 5mM β-mercaptoethanol) supplemented with PMSF and aprotinin. Cells were lysed by passage x 2 through a French press (12,000 lb/in^2^) and unbroken cells and insoluble aggregates were removed by centrifuging at 20,000 ×*g* for 30 min at 4°C. A majority of both PilB and PilT fusion proteins were soluble. Soluble fractions were incubated with Ni-NTA resin (Qiagen), washed per manufacturer’s recommendations, and eluted from the resin with 250 mM imidazole. Eluted protein was dialysed against Amylose resin binding buffer (20 mM Tris pH 8.0, 200 mM NaCl, 1 mM EDTA plus protease inhibitors). Dialysed proteins were bound to the amylose resin as per manufacture’s protocol (New England Biolabs) and eluted with binding buffer containing 10 mM Maltose. The two consecutive affinity purifications resulted in protein with > 90% purity as assessed by Coomassie staining.

Proteins were next run on a Size Exclusion Chromatography (SEC) column (Superdex 200 Increase, GE Healthcare) pre-equilibrated with 25 mM Tris pH 8.0, 250 mM NaCl, 5 mM MgCl2, 5 mM β-mercaptoethanol. Fractions eluting at a MW of ~700 kDa, correponding to the predicted mass of hexameric PilB or PilT, were collected, pooled, concentrated via Amicon (10 KDa cutoff) and used for further experiments. FimX and FimX(AAA) were purified as 6X-His tagged proteins as described previously [17].

### SEC binding assays

Hexameric PilB (50 μM, final) and either dimeric FimX or FimX(AAA) (50 μM, final) were combined in binding buffer (25 mM Tris pH 8, 150 mM NaCl, 5 mM MgCl_2_, 500 μM c-di-GMP). The reaction was incubated at RT for 30 minutes, then loaded onto a pre-equilibrated Superdex 200 10/300 gel filtration column (GE Healthcare). BioRad (#151–1901) molecular weight standard was run for reference. The column was run at 0.5 ml/min. After an isocratic flow of the buffer (3ml), the sample was loaded and the run continued for another 25 ml. Fractions of 0.5 ml were collected throughout the run. Collected fractions with OD_254_>baseline were analyzed by SDS-PAGE. Each protein was also individually analyzed by SEC under these conditions. The experiment was repeated twice with freshly purified protein each time.

### Luciferase complementation strain construction

The Rluc8 luciferase gene [61] was resynthesized by Genewiz, Inc. to reflect *P*. *aeruginosa* codon usage bias. The amino terminal 110 amino acids or the carboxy terminal 201 amino acids were amplified to generate Nluc and Cluc fragments; a flexible (GGGGS)_2_ linker sequence was introduced between the luciferase fragment and the bait/prey proteins during the cloning process. Fusion proteins were cloned under control of the arabinose inducible pBAD promoter in pMQ95 or a pMQ72 derivative (details available on request). Plasmids were introduced to *P*. *aeruginosa* by electroporation and transformants were selected on LB plates containing 50 μg ml^-1^ gentamicin and 100 μg ml^-1^ carbenicillin. For the experiments, the bacterial strains were grown under non-inducing (no arabinose) or inducing (0.05% arabinose) conditions.

### Split luciferase complementation assay

Luciferase activity was measured from plate grown bacteria. A bacterial colony was streaked heavily on LB or LB + 0.05% arabinose (with appropriate antibiotics) and the plates were grown for 14–16 hr at 37°C. Bacteria were then scraped off in PBS and normalized by OD_600_. 2 x10^8^ ml^-1^ bacterial cells were used per assay and 1 μM coelenterazine (Biotium, Hayward, CA) was delivered to each sample at the beginning of the measurement to initiate the bioluminescence reaction. Luminescence measurements were taken every 20 seconds for 1 minute to capture the peak of the reaction using a Tecan Infinite 200 PRO series. OD_600_ was also measured simultaneously for each sample. Relative Luciferase (LU/ OD_600_) was calculated for each sample. Each experiment was carried out 3–5 times on different days. The results presented here show a representative experiment.

### Surface pilin kinetic/ Pulse chase assay

Exponential-phase bacterial cultures (OD_600_ ~ 0.4–0.5) were vortexed for 2–3 minutes to shear off any assembled surface pili. Bacterial pellets were washed twice, then resuspended in fresh LB. An aliquot was taken at this time point (t = 0) before the cultures were re-incubated at 37°C with aeration. Aliquots were taken at specified time points (0.5, 1, 1.5, 2, 3, 4 h and overnight) and surface pilin was prepped from each sample as described previously [17]. Spent media was also collected at specific time points (0, 3 h, overnight) and TCA precipitated to account for any pili that might shear or be shed during bacterial culture. Surface pilin preps and the precipitated media samples were separated by SDS-PAGE and immuno-blotted using anti-PilA antiserum [52]. Samples were normalized to total protein (whole cell sample) at each time point.

### Cell fractionation

Bacterial cells were heavily grown on LB plates at 37°C for 12–14 h. Cells (normalized by OD_600_) were then scraped into resuspension buffer (0.1 M HEPES, pH 7.5 + 150 mM NaCl) containing protease inhibitors. The cell suspension was lysed by two passages through a French Press (14,000 psi). Unlysed bacteria were removed by centrifugation at 5000 x *g* for 10 minutes. The supernatant was then centrifuged at 20,000 x *g* for 30 minutes to separate membrane (pellet) and cytosol (supernatant). Pellets were resuspended in 1X SDS-loading buffer (to the same final volume as supernatant plus 2X SDS-loading buffer); all samples were subjected to SDS-PAGE and immunoblotting. To blot for PilQ, membrane pellets were first re-suspended in 10% SDS and boiled at 95°C for 10 minutes, and then boiled for another 10 minutes after addition of an equal volume of 2X SDS-loading buffer.

### Movie acquisition and analysis

Bacteria were cultured on LB agar plus antibiotics as appropriate (37°C x 16h, followed by 24h at RT) and single colonies were resuspended in 20 μl of ddH_2_O. Two μl of this suspension was spotted at the interface between a 1% Gro-Gellan agar pad and a MatTek 60 mm dish and incubated at 37°C for 15–30 minutes before imaging. Time-lapse movies (2–15 min) were captured using the 60x objective of a Volocity spinning disc confocal microscope equipped with an environmental chamber (LIVE CELL; Pathology Devices) and a Nikon Perfect Focus. Time-lapse videos were edited using Volocity and ImageJ. The cells on the images were outlined and the polar fluorescence intensities were obtained with MicrobeTracker software [62]. The presence and direction of cell motion were detected from MicrobeTracker data using a custom MATLAB script and were confirmed manually.

### Image acquisition and processing

Bacterial samples for FimX, PilT and PilB localization were prepared in a similar way as described above for live cell imaging except that slides were not incubated at 37°C prior to image acquisition. Each bacterial strain was freshly streaked from glycerol stock and imaged on at least 3 different days; multiple fields were captured from each same slide. All images were obtained at a 100X magnification using Nikon TE2000-S and processed via Slidebook 5.5.

To score distribution of fluorescent fusion proteins, cells with unipolar, bipolar or diffuse distribution were identified manually and counted using the cell counter plugin in ImageJ. A variation of < 20% was tolerated in polar fluorescence intensity signal for a cell to be scored as having a “bipolar” distribution of a fusion protein.

### Transmission electron microscopy

Bacteria in log phase grown in LB broth were allowed to bind to glow-discharged carbon grids for 1 min. Grids were stained twice with 1% phosphotungstate (1 min) and washed with water, then dried for 10–15 minutes before imaging on a Tecnai 12 Biotwin microscope (Center for Cell and Molecular Imaging, Yale University).

### Surface plasmon resonance

Surface plasmon resonance (SPR) experiments were performed using a BIAcore T100 instrument (GE Healthcare) at 25°C (Keck Foundation Biotechnology Resource Laboratory, Yale University). FimX and FimX(AAA) proteins were prepared for SPR measurements as described elsewhere [17] and immobilized on a CM5 sensor chip using a standard amine-coupling method. Series of two- and three-fold dilutions of c-di-GMP (0–300 nM) were injected onto the surface both sequentially and at random. Binding kinetics were evaluated using BIAevaluation software (GE Healthcare), and all sensograms were fitted using a 1:1 binding model.

## Supporting information

S1 Fig FimX localizes at the leading pole of a twitching bacteria. Image montage shows the time lapse of WT PA103Δ *fimX* expressing tdimer2-FimX under FimX promoter. Sequence shows a cell with enriched unipolar FimX at leading pole pulling another cell with FimX at lagging pole (arrow), resulting in movement of the latter.
(TIF)

S2 Fig Characterization of wild-type and mutant FimX constructs. (A) Sensograms of c-di-GMP binding to immobilized FimX or FimX(AAA) were obtained from surface plasmon resonance. Different concentrations of c-di-GMP (0–300 nM) are presented as an overlay plot aligned at the start of injection. K_D_ of 88 ± 5.6 nM for wild-type FimX was calculated using a simple 1:1 interaction model. The FimX(AAA) mutant shows no c-di-GMP binding under identical conditions. (B) Whole cell lysates from plate grown PA103 (WT) and isogenic Δ*fimX* bacteria expressing tdimer2 (52 kDa), tdimer2-FimX (128 kDa), tdimer2-FimXΔREC (119 kDa) and tdimer2-FimXΔEAL (103 kDa), as indicated. Whole cell lysates were prepared from plate-grown bacteria and normalized by total protein. Lysates were separated by 7.5% SDS-PAGE and immunoblotted with anti-RFP antiserum. Note the absence of “free” tdimer2 in bacteria expressing tdimer2-FimX fusions (lanes 3–5).
(TIF)

S3 Fig Expression levels of endogenous and plasmid-encoded FimX in different Pil mutants. Whole cell lysates of the PA14 transposon mutants without (A) or with a plasmid expressing tdimer2-FimX (B) were prepared from plate grown bacteria; samples were normalized by total protein. Lysates were separated by 10% SDS-PAGE and immunoblotted with anti-FimX antiserum (1:8000). Lanes are labeled with the site of each transposon insertion. Migration of molecular weight markers (kDa) is indicated for each panel.
(TIF)

S4 Fig Localization of FimX is altered in the absence of PilQ. (A) Western blot of the membrane (“P”, pellet) and cytosol (“S”, supernatant) fractions of PAO1 WT and PAO1Δ*pilQ*. Blots were probed with anti-FimX antiserum (1:8000; top panel) and anti-PilQ antiserum (1:1000; bottom panel). Anti-PilQ exhibits non-specific cross-reactivity with a more rapidly migrating band that served as a marker for the cytosolic compartment. (B) Subcellular distribution of FimX in PAO1 and PAO1Δ*pilQ*; > 250 cells were scored for each strain.
(TIF)

S5 Fig FimX is bipolar in absence of PilH. Images of WT PAO1 and PAO1Δ*pilH* expressing tdimer2-FimX.
(TIF)

S6 Fig Plasmid-borne YFP-PilB, but not chromosomal *attB*::GFP-PilB has a dominant negative effect on twitching. (A) Twitching zones of bacteria expressing plasmid-borne YFP-PilB or YFP-PilT compared to PAO1 plus empty vector. (B) Twitching zones of bacteria expressing GFP-PilB or GFP-PilT integrated at the *attB* site of PAO1. Bars show mean ± S.D. for 5–10 replicates.
(TIF)

S7 Fig Δ*fimX pilT* bacteria assemble type IV pili. Visualization of T4P by transmission electron microscopy. Bacteria from early exponential growth phase were stained directly with 1% phosphotungstate as described in Materials and Methods. Panels show a representative cell for each strain. Scale bar = 200 nm.
(TIF)

S8 Fig Bacteria require PilB to assemble pili. Western Blot analysis of surface pili (SP) and whole cell (WC) fractions of PA103 *ΔfimXΔpilB and ΔfimXΔpilTΔpilB* probed with anti-PilA antibody. Exponential growing bacteria were vortexed to shear off their pili (T0) and an aliquot was taken as different time points (0.5,1,1.5,2,3,4 and overnight) after growth in fresh media. The time points matched the points as in Fig 7.
(TIF)

S9 Fig FimX interacts with PilB in the absence of PilZ. Bar graph shows the relative luciferase units (LU/OD_600_) for FimX-PilB interaction in PAO1 and PAO1Δ*pilZ* and FimX-PilT interaction in PA103. Combination of the proteins or the vector control (V) was introduced in *P*. *aeruginosa* cells by electroporation and the strains were grown on LB plates (with appropriate antibiotics) for the luciferase complementation assay. FimX-FimX interaction was set to 100%.
(TIF)

S10 Fig FimX and PilB interact in *E*. *coli* as measured by split-luciferase assay. Bar graph shows the relative luciferase units (LU/OD_600_) for PilB interaction with FimX or AAA in *E*.*coli*. Combination of the proteins or the vector control (V) was introduced in *E*. *coli* by electroporation and the strains were grown on LB plates (with appropriate antibiotics and inducer) for the luciferase complementation assay.
(TIF)

S11 Fig PilB shows minimal ATPase activity in vitro that is not altered by addition of FimX. PilB, PilT and FimX were purified as described in Materials and Methods. (A) Coomassie stained gel showing purified proteins. “M” shows migration of Biorad Precision Plus Protein standards. (B) ATPase activity of purified PilB or PilT (10 or 20 μg) was assayed using Invitrogen EnzChek Phosphate Assay Kit. Protein was incubated for 5 minutes before addition of 1mM ATP. Hydrolysis was monitored at 360 nm using a Tecan Plate reader. An ATP-alone control was included and subtracted from the experimental wells. (C) ATPase activity of PilB, FimX or the two proteins incubated together in equimolar amounts was measured in the presence of 1 mM ATP. Phosphate release was monitored at 360 nm, as above. An ATP-alone blank was subtracted from all wells. Note the change in y-axis scale between panels B and C.
(TIF)

S1 Table Strains and plasmids used in the study. (DOCX)

S2 Table Primers used in the study. (DOCX)

S1 Movie Bacteria twitching with tdimer2-FimX at leading pole. Corresponds to Fig 1B.
(MOV)

S2 Movie Cell with bipolar FimX appears to be dragged. Corresponds to Fig 1F.
(MOV)

S3 Movie Movie corresponding to S1 Fig montage. (MOV)

S4 Movie FimX relocalizes during periods of bacterial movement and stalling. Corresponds to Fig 1D.
(MOV)
