# Supplementary figures and images for: Interaction of the cyclic-di-GMP binding protein FimX and the Type 4 pilus assembly ATPase promotes pilus assembly
Source: PLoS Pathog. 2017 Aug 30;13(8):e1006594. doi: 10.1371/journal.ppat.1006594 (PMC5595344; doi:10.1371/journal.ppat.1006594)

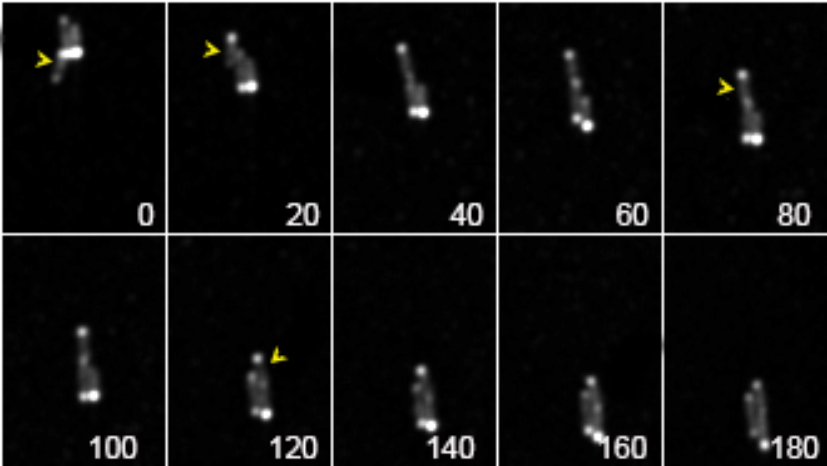

Supplement: S1 Fig — Image montage shows the time lapse of WT PA103Δ fimX expressing tdimer2-FimX under FimX promoter. Sequence shows a cell with enriched unipolar FimX at leading pole pulling another cell with FimX at lagging pole (arrow), resulting in movement of the latter. (TIF) [file ppat.1006594.s001.tif]

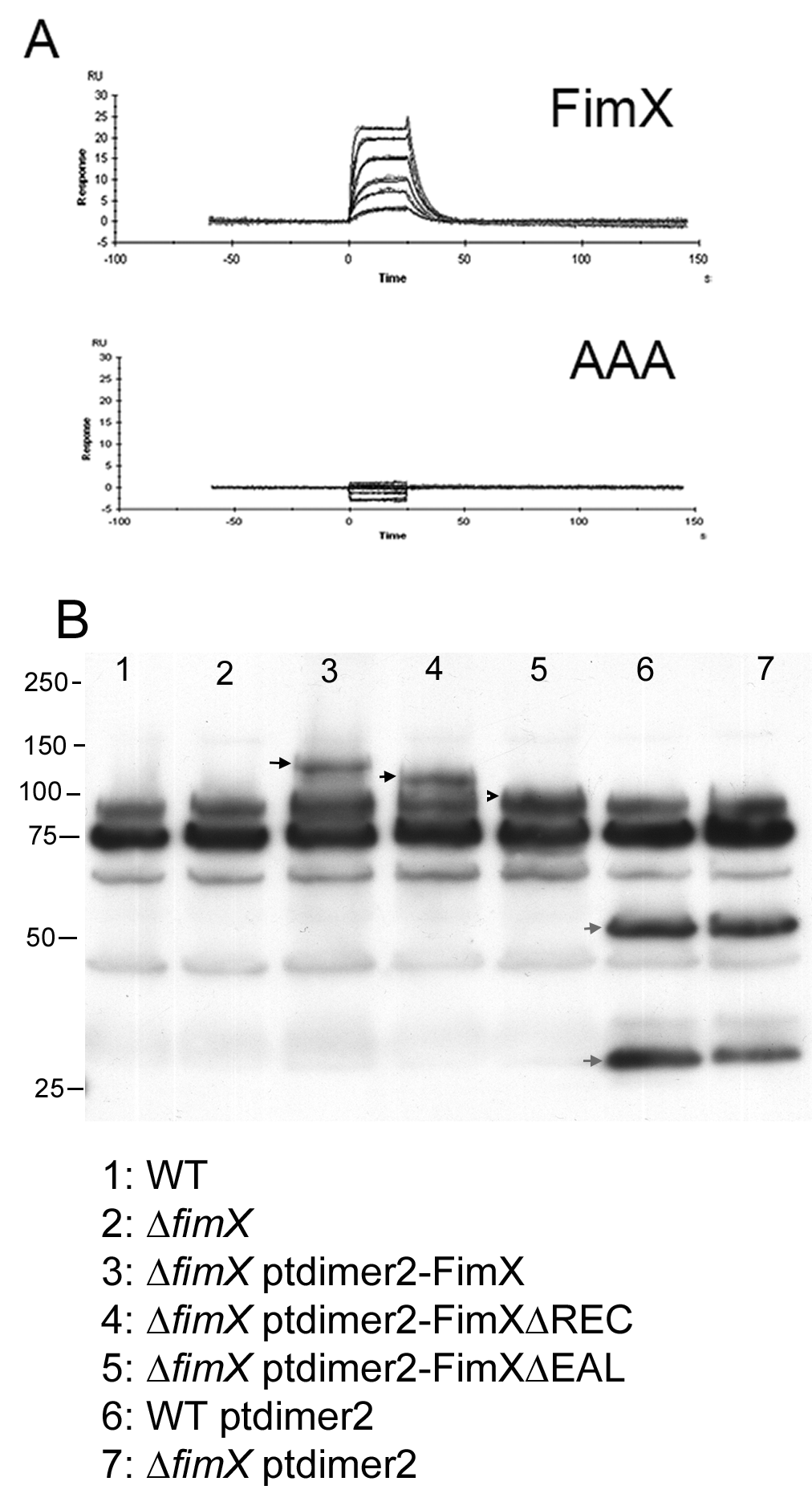

Supplement: S2 Fig — (A) Sensograms of c-di-GMP binding to immobilized FimX or FimX(AAA) were obtained from surface plasmon resonance. Different concentrations of c-di-GMP (0–300 nM) are presented as an overlay plot aligned at the start of injection. KD of 88 ± 5.6 nM for wild-type FimX was calculated using a simple 1:1 interaction model. The FimX(AAA) mutant shows no c-di-GMP binding under identical conditions. (B) Whole cell lysates from plate grown PA103 (WT) and isogenic ΔfimX bacteria expressing tdimer2 (52 kDa), tdimer2-FimX (128 kDa), tdimer2-FimXΔREC (119 kDa) and tdimer2-FimXΔEAL (103 kDa), as indicated. Whole cell lysates were prepared from plate-grown bacteria and normalized by total protein. Lysates were separated by 7.5% SDS-PAGE and immunoblotted with anti-RFP antiserum. Note the absence of “free” tdimer2 in bacteria expressing tdimer2-FimX fusions (lanes 3–5). (TIF) [file ppat.1006594.s002.tif]

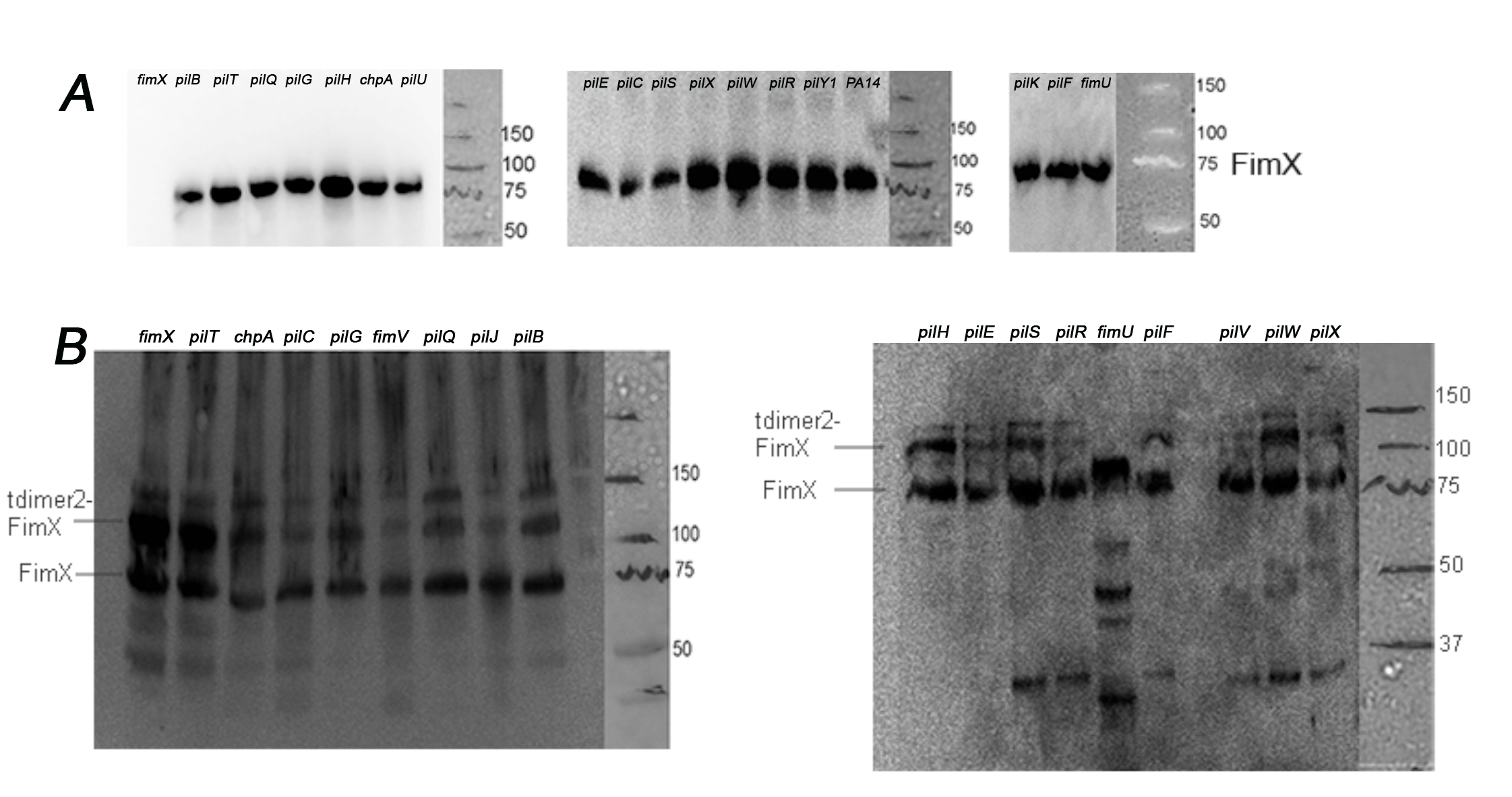

Supplement: S3 Fig — Whole cell lysates of the PA14 transposon mutants without (A) or with a plasmid expressing tdimer2-FimX (B) were prepared from plate grown bacteria; samples were normalized by total protein. Lysates were separated by 10% SDS-PAGE and immunoblotted with anti-FimX antiserum (1:8000). Lanes are labeled with the site of each transposon insertion. Migration of molecular weight markers (kDa) is indicated for each panel. (TIF) [file ppat.1006594.s003.tif]

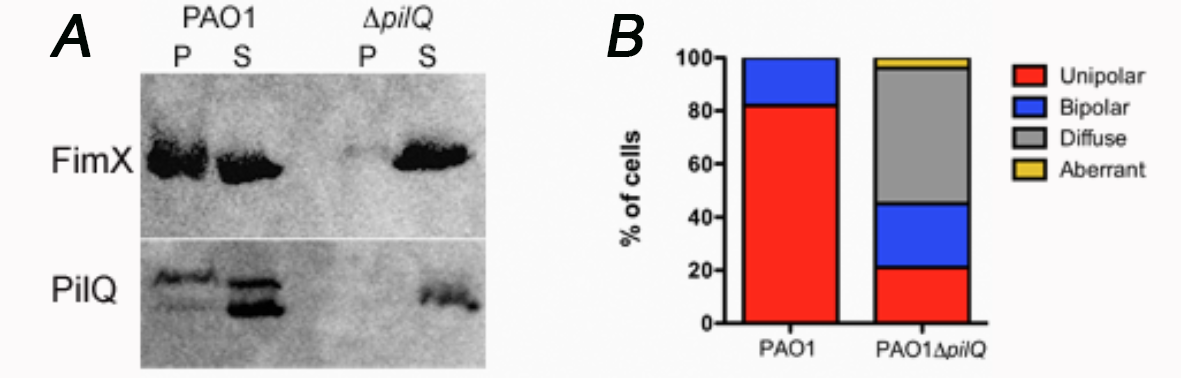

Supplement: S4 Fig — (A) Western blot of the membrane (“P”, pellet) and cytosol (“S”, supernatant) fractions of PAO1 WT and PAO1ΔpilQ. Blots were probed with anti-FimX antiserum (1:8000; top panel) and anti-PilQ antiserum (1:1000; bottom panel). Anti-PilQ exhibits non-specific cross-reactivity with a more rapidly migrating band that served as a marker for the cytosolic compartment. (B) Subcellular distribution of FimX in PAO1 and PAO1ΔpilQ; > 250 cells were scored for each strain. (TIF) [file ppat.1006594.s004.tif]

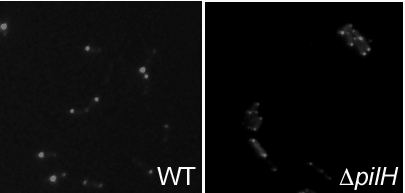

Supplement: S5 Fig — Images of WT PAO1 and PAO1ΔpilH expressing tdimer2-FimX. (TIF) [file ppat.1006594.s005.tif]

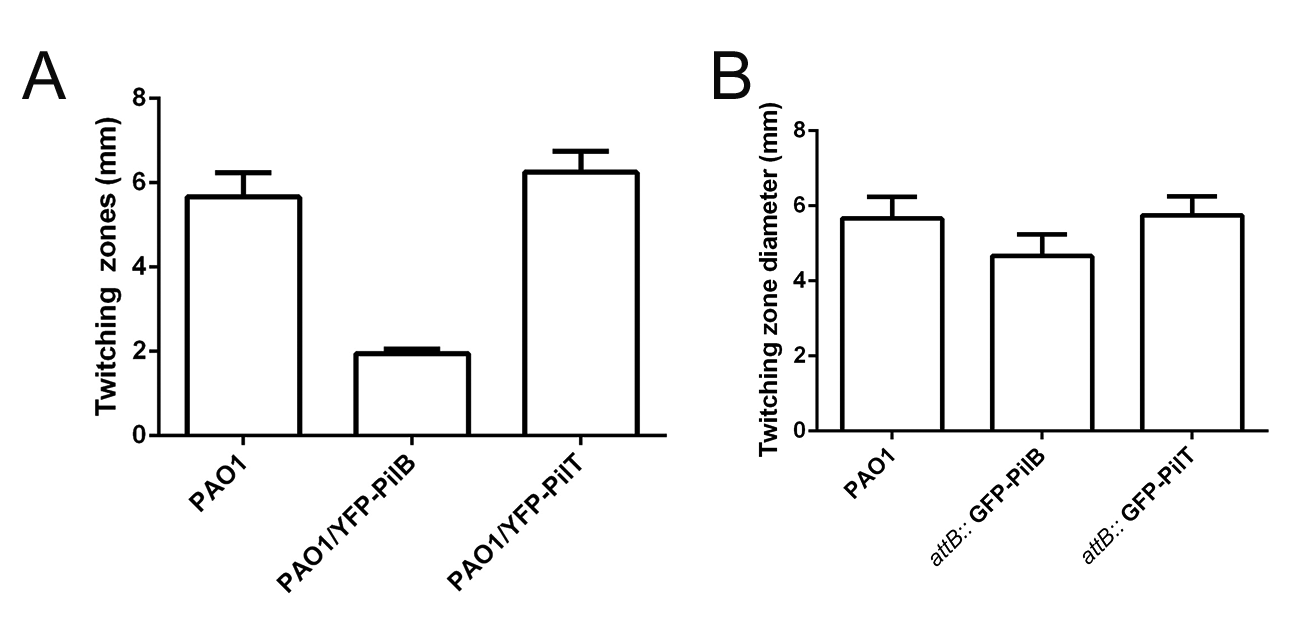

Supplement: S6 Fig — (A) Twitching zones of bacteria expressing plasmid-borne YFP-PilB or YFP-PilT compared to PAO1 plus empty vector. (B) Twitching zones of bacteria expressing GFP-PilB or GFP-PilT integrated at the attB site of PAO1. Bars show mean ± S.D. for 5–10 replicates. (TIF) [file ppat.1006594.s006.tif]

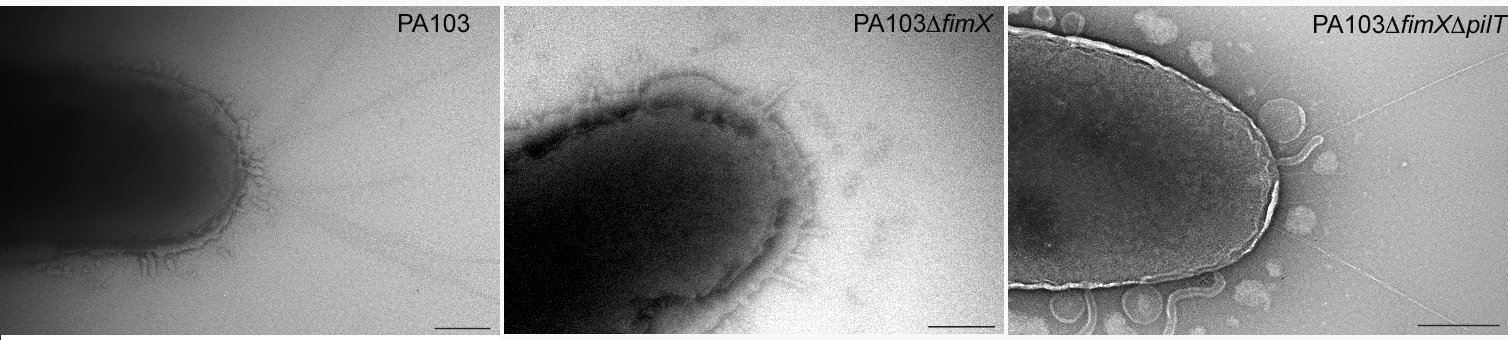

Supplement: S7 Fig — Visualization of T4P by transmission electron microscopy. Bacteria from early exponential growth phase were stained directly with 1% phosphotungstate as described in Materials and Methods. Panels show a representative cell for each strain. Scale bar = 200 nm. (TIF) [file ppat.1006594.s007.tif]

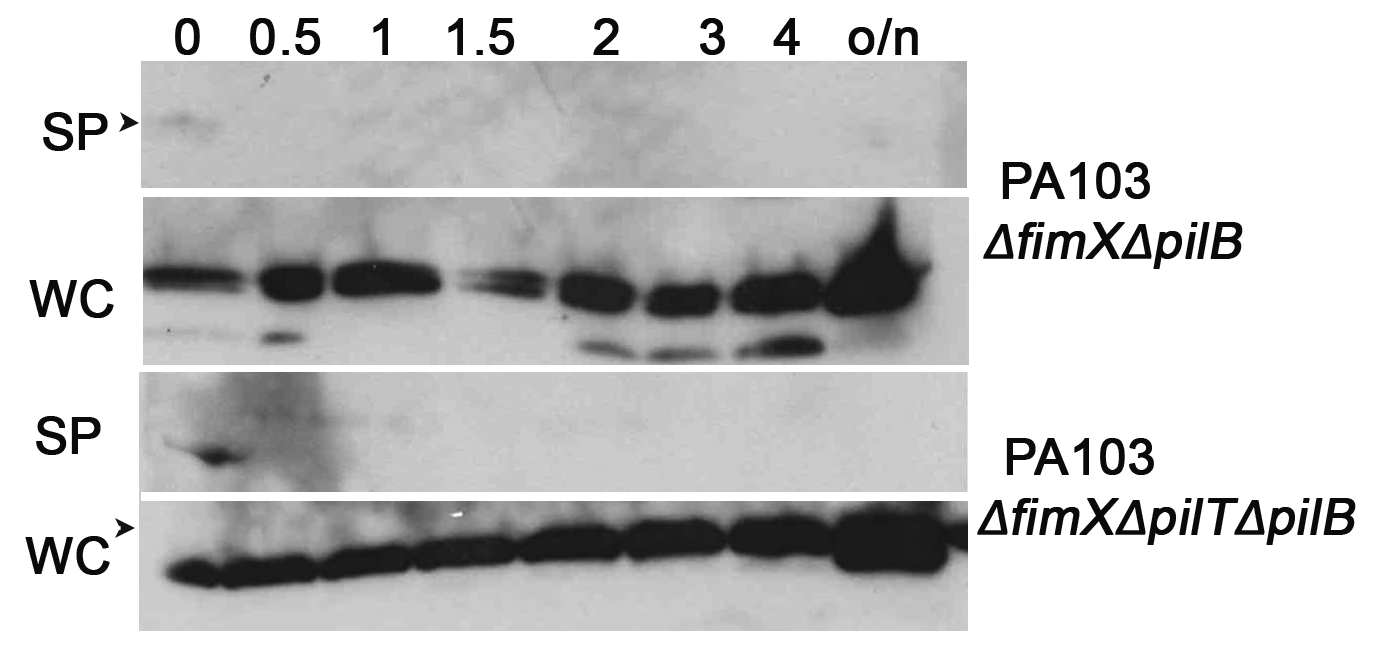

Supplement: S8 Fig — Western Blot analysis of surface pili (SP) and whole cell (WC) fractions of PA103 ΔfimXΔpilB and ΔfimXΔpilTΔpilB probed with anti-PilA antibody. Exponential growing bacteria were vortexed to shear off their pili (T0) and an aliquot was taken as different time points (0.5,1,1.5,2,3,4 and overnight) after growth in fresh media. The time points matched the points as in Fig 7. (TIF) [file ppat.1006594.s008.tif]

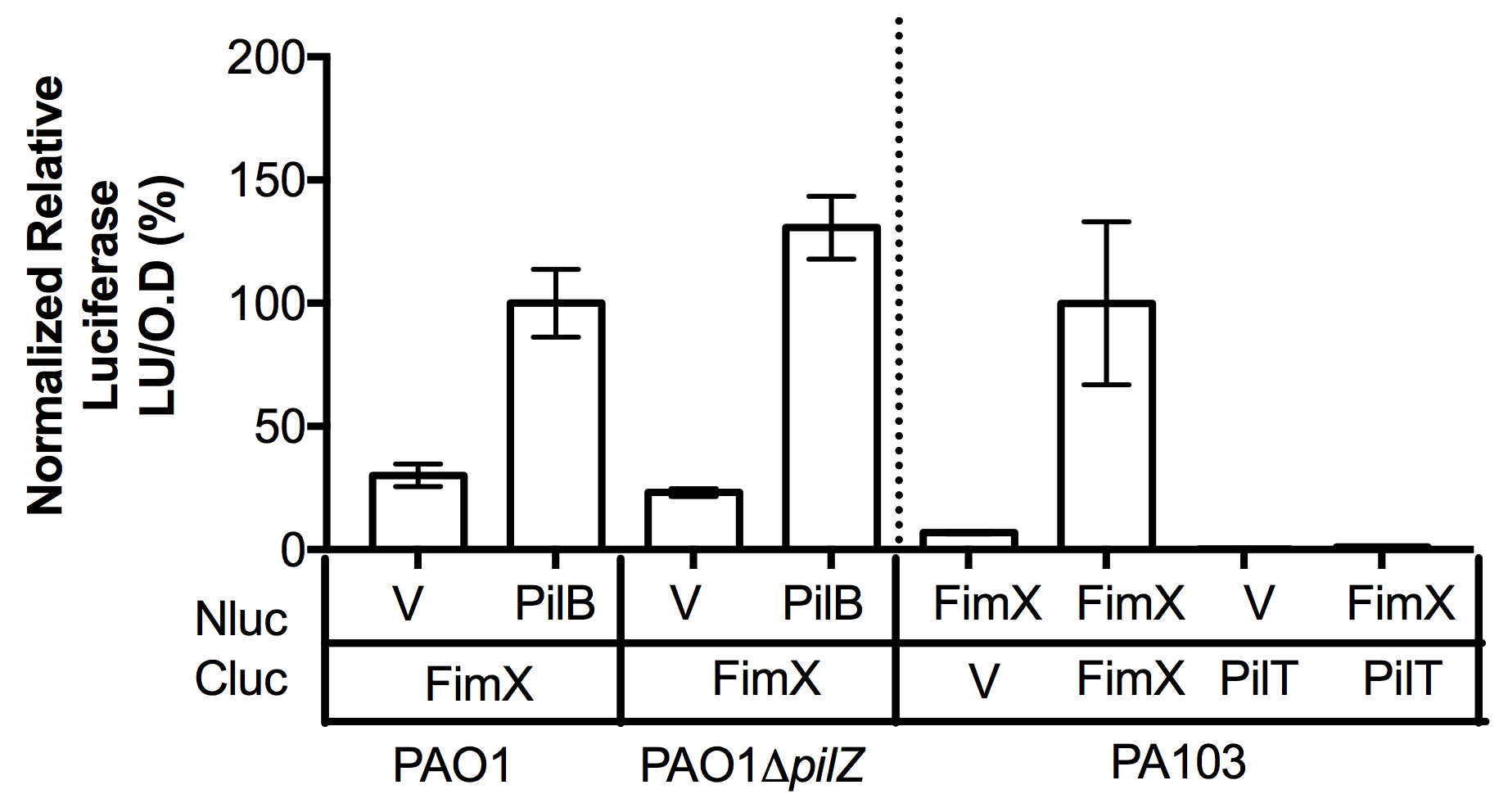

Supplement: S9 Fig — Bar graph shows the relative luciferase units (LU/OD600) for FimX-PilB interaction in PAO1 and PAO1ΔpilZ and FimX-PilT interaction in PA103. Combination of the proteins or the vector control (V) was introduced in P. aeruginosa cells by electroporation and the strains were grown on LB plates (with appropriate antibiotics) for the luciferase complementation assay. FimX-FimX interaction was set to 100%. (TIF) [file ppat.1006594.s009.tif]

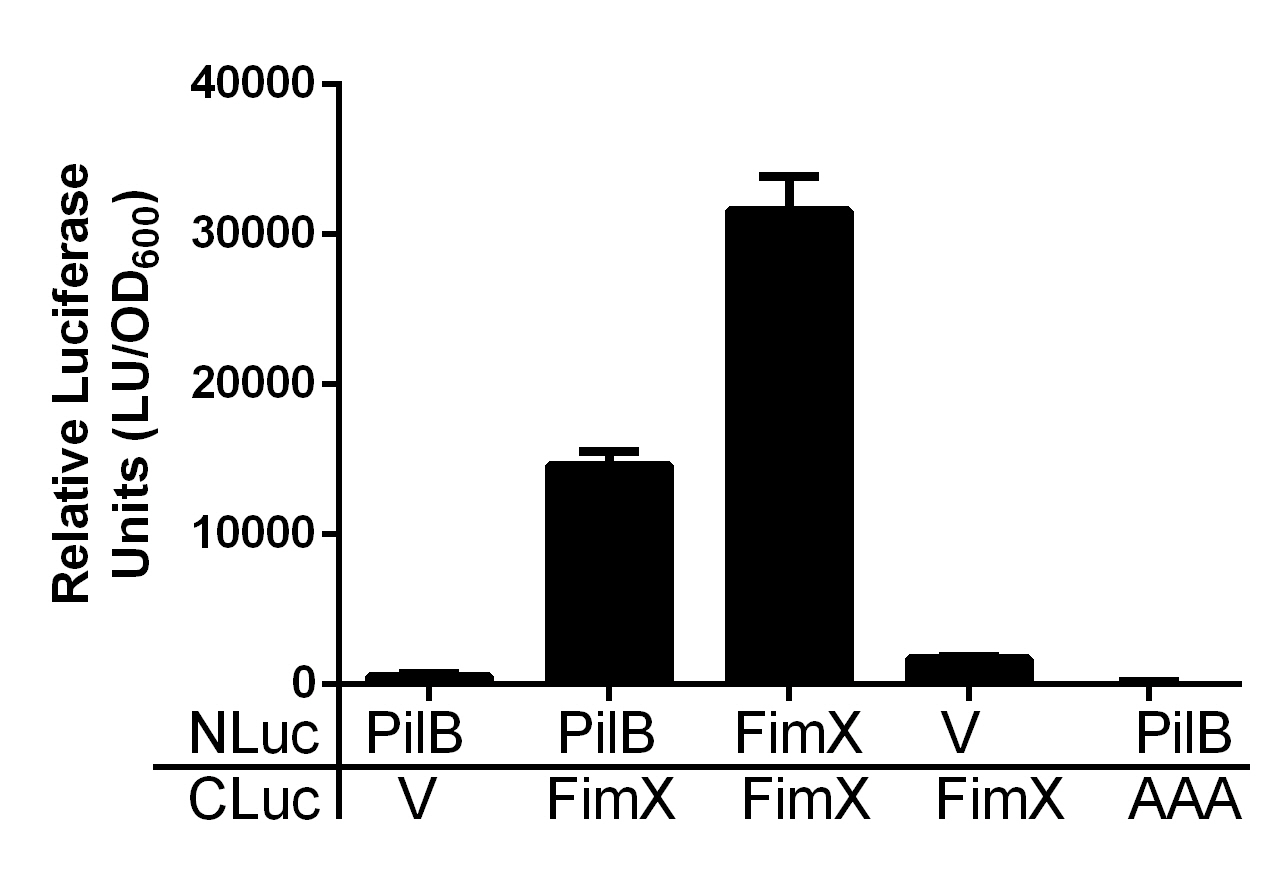

Supplement: S10 Fig — Bar graph shows the relative luciferase units (LU/OD600) for PilB interaction with FimX or AAA in E.coli. Combination of the proteins or the vector control (V) was introduced in E. coli by electroporation and the strains were grown on LB plates (with appropriate antibiotics and inducer) for the luciferase complementation assay. (TIF) [file ppat.1006594.s010.tif]

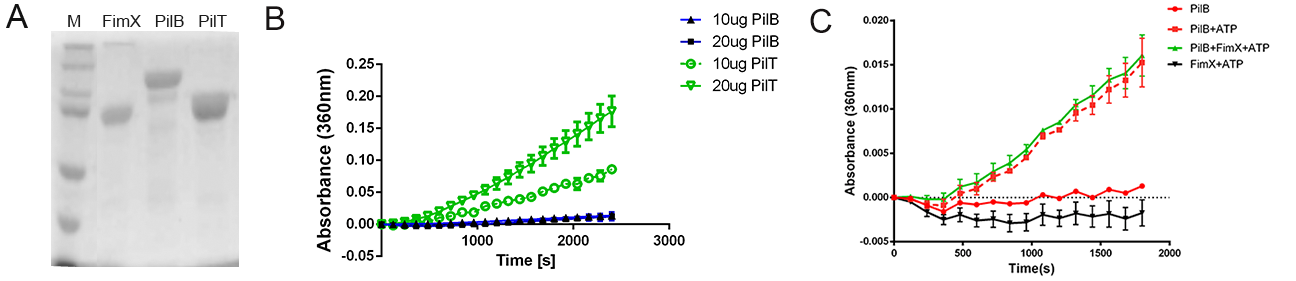

Supplement: S11 Fig — PilB, PilT and FimX were purified as described in Materials and Methods. (A) Coomassie stained gel showing purified proteins. “M” shows migration of Biorad Precision Plus Protein standards. (B) ATPase activity of purified PilB or PilT (10 or 20 μg) was assayed using Invitrogen EnzChek Phosphate Assay Kit. Protein was incubated for 5 minutes before addition of 1mM ATP. Hydrolysis was monitored at 360 nm using a Tecan Plate reader. An ATP-alone control was included and subtracted from the experimental wells. (C) ATPase activity of PilB, FimX or the two proteins incubated together in equimolar amounts was measured in the presence of 1 mM ATP. Phosphate release was monitored at 360 nm, as above. An ATP-alone blank was subtracted from all wells. Note the change in y-axis scale between panels B and C. (TIF) [file ppat.1006594.s011.tif]
