# Supplementary material for: Interaction of the cyclic-di-GMP binding protein FimX and the Type 4 pilus assembly ATPase promotes pilus assembly
Source: PLoS Pathog. 2017 Aug 30;13(8):e1006594. doi: 10.1371/journal.ppat.1006594 (PMC5595344; doi:10.1371/journal.ppat.1006594)
Supplement: S1 Table — (DOCX) [file ppat.1006594.s012.docx]

**S1 Table. Strains and Plasmids used in the study**

| **Strain or Plasmids** | **Genotype or description** | **Reference** |
| --- | --- | --- |
| *E. coli* strains |  |  |
| DH5α | *supE44* Δ*lacU169*(Φ80d*lacZ*ΔM15)  *hsdR17 thi-1 relA1 recA1* | Invitrogen |
| S17.1 | Used for mating constructs into *P. aeruginosa*; *thi pro hsdR recA* RP4-2 (Tc::Mu) (Km::Tn*7*) | [62] |
| *P. aeruginosa* strains |  |  |
| PA103 | Wild type; Aflagellate virulent lung isolate of *P. aeruginosa* | [63] |
| PAO1 | Wild type | [64] |
| PA14 *fimX*::Tn | Transposon insertion in *fimX* | George O’ Toole |
| PA14 *pilQ*::Tn | Transposon insertion in *pilQ* | George O’ Toole |
| PA14 *pilF*::Tn | Transposon insertion in *pilF* | George O’ Toole |
| PA14 *pilC*::Tn | Transposon insertion in *pilC* | George O’ Toole |
| PA14 *pilB*::Tn | Transposon insertion in *pilB* | George O’ Toole |
| PA14 *pilT*::Tn | Transposon insertion in *pilT* | George O’ Toole |
| PA14 *pilU*::Tn | Transposon insertion in *pilU* | George O’ Toole |
| PA14 *pilH*::Tn | Transposon insertion in *pilH* | George O’ Toole |
| PA14 *pilK*::Tn | Transposon insertion in *pilK* | George O’ Toole |
| PA14 *pilG*::Tn | Transposon insertion in *pilG* | George O’ Toole |
| PA14 *chpA*::Tn | Transposon insertion in *chpA* | George O’ Toole |
| PA14 *fimU*::Tn | Transposon insertion in *fimU* | George O’ Toole |
| PA14 *pilX*::Tn | Transposon insertion in *pilX* | George O’ Toole |
| PA14 *pilW*::Tn | Transposon insertion in *pilW* | George O’ Toole |
| PA14 *pilJ*::Tn | Transposon insertion in *pilJ* | George O’ Toole |
| PA14 *pilI*::Tn | Transposon insertion in *pilI* | George O’ Toole |
| PA14 *pilE*::Tn | Transposon insertion in *pile* | George O’ Toole |
| PA14 *pilS*::Tn | Transposon insertion in *pilS* | George O’ Toole |
| PA14 *pilR*::Tn | Transposon insertion in *pilR* | George O’ Toole |
| PA14 *pilV*::Tn | Transposon insertion in *pilV* | George O’ Toole |
| PA103 Δ*pilB* | PA103 containing an in frame deletion of aa 4-563 of PilB | This study |
| PA103Δ*fimX* | PA103 containing an in frame deletion of aa 15-685 of FimX | [17] |
| PA103 Δ *fimX*Δ*pilT* | PA103 containing an in frame deletion of PilT and aa 15-685 of FimX | This study |
| PA103Δ*fimX*Δ*pilT*Δ*pilB* | PA103 containing an in frame deletion of PilT, aa 15-685 of FimX and aa 4-563 of PilB | This study |
| PA103Δ*fimX*Δ*pilT*  *attB*::FimX | WT FimX under *fimX* promoter integrated at the *attB* site in PA103Δ *fimX* Δ*pilT* background | This study |
| PA103Δ*fimX*Δ*pilT*  *attB*::FimX (AAA) | Point mutant FimX (AAA) under *fimX* promoter integrated at the *attB* site in PA103Δ*fimX* Δ*pilT* background | This study |
| PA103Δ*fimX*Δ*pilT*  *attB*::FimX ΔEAL | Deletion mutant FimX (ΔEAL) under *fimX* promoter integrated at the *attB* site in PA103Δ*fimX* Δ*pilT* background | This study |
| PAO1Δ*pilQ* | PAO1 containing an in frame deletion of aa 7-707 of PilQ | This study |
| PAO1Δ*pilH* | PAO1 containing an in frame deletion of aa 4-118 of PilH | This study |
| PAO1Δ*pilH*  *attB*::PilH | WT PilH under its own promoter integrated at the *attB* site in PAO1Δ*pilH* background | This study |
| PAO1Δ*pilZ* | PAO1 containing an in frame deletion of aa 1-352 of PilZ | This study |
| PA103 Δ*pilT* | PA103 containing an in frame deletion of PilT | This study |
| PA103*attB:: P_pilT_-*GFP-PilT | PA103 containing GFP-PilT at the *attB* site on the chromosome | This study |
| PA103*attB::P_pilB_-GFP*-PilB | PA103 containing GFP-PilB at the *attB* site on the chromosome | This study |
| PA103Δ*fimX attB:: P_pilB_-*GFP-PilB | PA103 Δ*fimX* containing GFP-PilB at the *attB* site on the chromosome | This study |
| *Plasmids* |  |  |
| pUCP-SK | Constitutive expression vector in *P. aeruginosa* under *plac,* Cb^r^ | [65] |
| ptdimer2-FimX | Tdimer2-FimX under *fimX* promoter in high copy pUCP-KS vector, Cb^r^ | [17] |
| pMQ95 | Arabinose inducible expression vector under pBAD, Cb^r^ | [66] |
| pMQ72 | Arabinose inducible expression vector under pBAD, Gm^r^ | [66] |
| NLuc | N-terminal 110 amino acid of RLuc cloned in pMQ95 | This study |
| CLuc | C-terminal 201 amino acid of RLuc cloned in pMQ72 | This study |
| pEX18 | Allelic replacement suicide plasmid; Gm^r^ *sacB oriT* | [59] |
| miniCTX-2 | Contains *attP* site for integration at chromosomal *attB site*; Tc^r^ | [67] |
| pFLP2 | Source of inducible *flp* recombinase; Ap^r^ (Cb^r^) | [59] |
| pVL847 | E.coli expression vector, Gm^r^ | Vincent Lee |
| YFP-PilB | YFP-PilT under p*lac* in high copy pUCP20, Gm^r^ | [32] |
| YFP-PilT | YFP-PilB under p*lac* in high copy pUCP20, Gm^r^ | [32] |
| GFP-PilB | GFP-PilB under PilB promoter (500 bp upstream region of PilB) in miniCTX-2, Tc^r^ | This study |
| GFP-PilT | GFP-PilB under PilT promoter (500 bp upstream region of PilT) in miniCTX-2, Tc^r^ | This study |

**Abbreviations used:** Tc, tetracycline; Km, kanamycin; Cb, carbenicillin; Gm, gentamicin; Ap, ampicillin
