# Supplementary material for: Interaction of the cyclic-di-GMP binding protein FimX and the Type 4 pilus assembly ATPase promotes pilus assembly
Source: PLoS Pathog. 2017 Aug 30;13(8):e1006594. doi: 10.1371/journal.ppat.1006594 (PMC5595344; doi:10.1371/journal.ppat.1006594)
Supplement: S2 Table — (DOCX) [file ppat.1006594.s013.docx]

**S2 Table. Primers used in the study**

| **Primer Name** | SEQUENCE (5’-3’) |
| --- | --- |
| PilQN1 | CACCGAGGTACCCGGGCTGCTCGAGGACATCAC |
| PilQN2 | GAAGGATCC CGAGAGGCCACTGTTCATCGTCCGAC |
| PilQC1 | GGAGGATCCGCCATCGCAATCGGTCGCTGATATCG |
| PilQC2 | CCGAGGAGTCGACCTGGGACAACAGGGTGGTCGGC |
| PilHN1 | GCCTTCTGGTAGAGCTCCTGCTGTTCCATGTAG |
| PilHN2 | GAGGGTACCACGAGCCATGGGATCCCCATCACG |
| PilHC1 | GAGGGTACCGCGGGCTGAGCCCGTCGCAGCGCATC |
| PilHC2 | GCGCATCCTTCAGAAGCTTGAACGCCTCGCCCTTGC |
| PilTN1 | GCGGTACCGATGCGGACCCAGGTCGCACCTTC |
| PilTN2 | GGCGGAGAGGTGGAGCTCCGAAGCGCCCTG |
| PilTC1 | GATCAGCCGCGAGAACGCCCGCGAGAAG |
| PilTC2 | GGTCTCCCGCAAGCTTACCTCGCCGATC |
| PilZN1 | CAGAATTCCGCCCAGCACGCCCACGC |
| PilZN2 | GGGATCCGCGGCCGCCAGATTGGGTGGCAAACTCAT |
| PilZC1 | GAGGATCCGCGGCCGCACACGATGTAACGCTAGACAGG |
| PilZC2 | GAAAGCTTCCCCCCAAGTACCCAGCCAG |
| PilBN1 | GGGAATTCCCTTCTCATTACGAATGAGCTGCTC |
| PilBN2 | CGCCATGGATTAATCCTTGGTCGTCGTTCATGGGGAAGGAATC |
| PilBC1 | GATTCCTTCCCCATGAACGACGACCAAGGATTAATCCATGGCG |
| PilBC2 | GGAAGCTTGGTCCGTGCATAACGGGCGACC |
| PilBG1 | CCCCCGGGCTGCAGGAATTCCCCAACTTGTTGGCATCCGGCT |
| PilBG2 | CCTTTACTCATGGGGAAGGAATCGCAGAAGGGCTATTG |
| PilBG3 | TCCTTCCCCATGAGTAAAGGAGAAGAACTTTTCACTGG |
| PilBG4 | GCGGCGCCGCCTTTGTATAGTTCATCCATGCCATGTGTAATC |
| PilBG5 | TATACAAAGGCGGCGCCGCCGGCGGCATGAACGACAGCATC |
| PilBG6 | GGGCGAATTGGGTACCTTAATCCTTGGTCACGCGGTTGACTTCCTCCAG |
| PilTG1 | CCGCTCTAGAACTAGTGGATCCACTGGAAATGCTCGGCGATGGG |
| PilTG2 | TTTACTCATGGGACTCCCCAATTACAAGCAAGCA |
| PilTG3 | GGGAGTCCCATGAGTAAAGGAGAAGAACTTTTCACTGG |
| PilTG4 | GCGGCGCCGCCTTTGTATAGTTCATCCATGCCA |
| PilTG5 | TATACAAAGGCGGCGCCGCCGGCGGCATGGATATTACCGAGCTGCTC |
| PilTG6 | GGGCGAATTGGGTACCTCAGAAGTTTTCCGGGATCTTCGCCTTCTCG |
| NLuc-F | GTCGACGGAGATATACATGACCTCAGGAATCTACGATC |
| NLuc-R | ACTAGTGCGGCCGCCGAGCCACCGCCACCCAAGTTCAGGAGTTCGAACCA |
| CLuc-F | CAATTGGAGATATACATGCCCAAGAAGATCATTTTCGTCGG |
| CLuc-R | CCCGGGCGGCCGCCGAGCCACCGCCACCCTGTTCGTTCTTGAGCACGCG |
| NLucPilB-F | GACGCGGCCGCAATGAACGACAGCATCCAACTGAGC |
| NLucPilB-R | GACGCGGCCGCTTAATCCTTGGTCACGCGGTTGAC |
| CLucPilT-F | GACGCGGCCGCA ATGGATATTACCGAGCTGCTCGCC |
| CLucPilT-R | GCGAAGCTTTCAGAAGTTTTCCGGGATCTTCGCCTT |
| CLucFimX-F | GGGCGGCCGCAATGGCCATCGAAAAGAAAACC |
| CLucFimX-R | GCGCAAGCTTTCATTCGTCTCCCGAGGAGAAGTCG |
| YFP-F(EcoR1) | AAAAGAATTCAGGAGGACAGCTATGGTGAGCAAGGGCG |
| YFP-R(SacI) | GCCGAGCTCCTTGTACAGCTCGTCCATGCCGAG |
| PilB-F(SacI) | GCCGAGCTCATGAACGACAGCATCCAACTGAGCGG |
| PilB-R(Kpn1) | GCGGTACCTTAATCCTTGGTCACGCGGT |
| YFP-F(SacI) | AAGAGCTCAGGAGGACAGCTATGGTGAGCAAGGGCGAGGAG |
| PilT-F(SacI) | GCCGAGCTCATGGATATTACCGAGCTGCTCGCC |
| PilT-R(HindIII) | ATTAAGCTTTCAGAAGTTTTCCGGGATCTTCGCC |
| PilHupF | GCGAATTCTTATGGATAGAGTTGCGTCGAGTGTGC |
| PilHR | GCAAGCTTGCGATGCGCTGCGACGGGCTCA |
